# Pragmatic, open-label, multicentre, randomised controlled trial to guide initial therapy for immune checkpoint inhibitor-induced inflammatory arthritis comparing standard of care (prednisolone) to adalimumab without glucocorticoids: REACT trial protocol

**DOI:** 10.1136/bmjopen-2026-116847

**Published:** 2026-03-03

**Authors:** Benjamin A Fisher, Anna Rowe, Chris Hodson, Manpreet Wilkhu, Emily Williams, Elliot Turner, Andrew Allard, Tim Blake, Michele Bombardieri, Andrew P Cope, Shirish Dubey, Kulveer Mankia, Tamir Malley, Owen Moore, Miranda Payne, Ruth Plummer, Michael Tilby, Tania Tillett, Ernest Wong, Yin Wu, Andrew Filer, Arthur Pratt, Lalit Pallan, Victoria Homer

**Affiliations:** 1 School of Infection, Inflammation and Immunology, University of Birmingham, Birmingham, UK; 2 Cancer Research UK Clinical Trials Unit, University of Birmingham, Birmingham, UK; 3 NIHR Birmingham Biomedical Research Centre, Birmingham, UK; 4 Patient partner, Birmingham, UK; 5 Royal National Hospital for Rheumatic Diseases, Royal United Hospitals Bath NHS Foundation Trust, Bath, UK; 6 Rheumatology Department, University Hospitals Coventry and Warwickshire NHS Trust, Coventry, UK; 7 Centre for Experimental Medicine and Rheumatology, Queen Mary University of London, London, UK; 8 Centre for Rheumatic Diseases, School of Immunology and Microbial Sciences, Faculty of Life Sciences and Medicine, King’s College London, London, UK; 9 Nuffield Orthopaedic Centre, Oxford University Hospitals NHS Foundation Trust, Oxford, UK; 10 Nuffield Department of Orthopaedics, Rheumatology and Musculoskeletal Sciences, University of Oxford, Oxford, UK; 11 Leeds Institute of Rheumatic and Musculoskeletal Medicine, University of Leeds, Leeds, UK; 12 NIHR Leeds Biomedical Research Centre, Leeds Teaching Hospitals NHS Trust, Leeds, UK; 13 Rheumatology Department, Royal Free Hospital, London, UK; 14 Department of Rheumatology, Derriford Hospital, University Hospitals Plymouth NHS Trust, Plymouth, UK; 15 Oxford Cancer Centre, Oxford University Hospitals NHS Foundation Trust, Oxford, UK; 16 Sir Bobby Robson Cancer Trials Research Centre, Newcastle Hospitals NHS Foundation Trust, Newcastle upon Tyne, UK; 17 Translational and Clinical Research Institute, Newcastle University, Newcastle upon Tyne, UK; 18 Oncology Department, University Hospitals Coventry and Warwickshire NHS Trust, Coventry, UK; 19 Oncology Department, Royal United Hospitals Bath NHS Foundation Trust, Bath, UK; 20 Rheumatology Department, Queen Alexandra Hospital, Portsmouth University Hospital NHS Trust, Portsmouth, UK; 21 Centre for Inflammation Biology and Cancer Immunology, King’s College London, London, UK; 22 Department of Medical Oncology, Guy's and St Thomas' Hospitals NHS Foundation Trust, London, UK; 23 Musculoskeletal Unit, Newcastle Upon Tyne Hospitals NHS Foundation Trust, Newcastle Upon Tyne, UK; 24 NIHR Newcastle Biomedical Research Centre, Newcastle upon Tyne, UK; 25 Department of Oncology, University Hospitals Birmingham NHS Foundation Trust, Birmingham, UK

**Keywords:** Clinical Trial, Drug Therapy, IMMUNOLOGY, RHEUMATOLOGY, ONCOLOGY

## Abstract

**Introduction:**

Immune checkpoint inhibitors (ICIs) have revolutionised cancer treatment through targeted disruption of the physiological pathways that maintain tissue tolerance, but which are co-opted by cancers to evade immunosurveillance. Thus, the resultant T-cell activity often causes immune-related adverse events including immune checkpoint inhibitor-induced inflammatory arthritis (ICI-IA). ICI-IA results in functional impairment that frequently persists, even after ICI discontinuation, with substantial quality-of-life impacts for cancer survivors.

A high-quality body of evidence to guide ICI-IA management remains an unmet need. Pharmacological treatment may be prolonged, typically begins with non-specific immunosuppression, including systemic steroids, and is usually only rationalised to more targeted therapy in resistant cases. Moreover, retrospective data suggest the high dose glucocorticoids sometimes used in new-onset ICI-IA may be associated with worse cancer outcomes.

Tumour necrosis factor (TNF) inhibition strategies are well established with excellent efficacy and safety profiles in ‘spontaneous’ inflammatory arthritides including rheumatoid and psoriatic arthritis. Mechanistic evidence from ex vivo and murine studies also supports the utility of anti-TNF therapy for steroid-refractory cases of ICI-IA. Although good clinical responses have been reported in this setting, the REACT trial (REmission induction of Arthritis caused by Cancer ImmunoTherapy) aims to provide randomised and robust clinical evidence for deploying targeted therapy earlier in ICI-IA management. It will test whether up-front anti-TNF therapy can more effectively and quickly control symptoms, reduce glucocorticoid exposure, prevent early ICI discontinuation and increase the frequency of drug-free ICI-IA remission.

**Methods and analysis:**

REACT is a prospective, multicentre, open-label, superiority, two-arm, randomised controlled clinical trial to guide initial therapy for patients with ICI-IA. The trial will compare the current standard of care (initial prednisolone; Arm A) with the anti-TNF drug, adalimumab without glucocorticoids (Arm B).

The primary outcome is glucocorticoid-free arthritis remission rate at 24 weeks where remission is defined as: (i) No use of systemic or intra-articular glucocorticoids (except when used for adrenal insufficiency) within 4 weeks prior to assessment at 24 weeks; and (ii) absence of synovitis on clinical examination.

**Ethics and dissemination:**

The protocol was approved by East Midlands—Leicester South Research Ethics Committee on 31-Oct-2024 (Ref: 24/EM/0202). Participants are required to provide written informed consent. The results of this trial will be disseminated through national and international presentations and peer-reviewed publications.

**Trial registration number:**

ISRCTN18217497.

**STRENGTHS AND LIMITATIONS OF THIS STUDY**

- REACT tests the most widely used anti-TNF agent in the UK, adalimumab, which has the convenience of subcutaneous administration.
- Adalimumab is being used without glucocorticoids, avoiding unnecessary over-immunosuppression.
- The lack of placebo reduces burden, complexity and cost of the trial.
- REACT has been co-developed with patient partners who co-designed the patient-centred ranked composite outcome that will be used as an exploratory outcome.
- REACT does not use loading doses of adalimumab, which have been shown to reach steady state drug levels more quickly and more rapidly control inflammation.

## Introduction

 Immune checkpoint inhibitors (ICIs) have revolutionised cancer treatment through targeted disruption of the physiological pathways that maintain tissue tolerance, but which are co-opted by cancers to evade immunosurveillance. However, the heightened T cell activity that results also causes immune-related adverse events (IrAEs).^1^,^3^ Immune checkpoint inhibitor-induced inflammatory arthritis (ICI-IA) occurs in 5–7% of treated patients.^2^ The indications for ICI therapy are rapidly increasing (projected compound annual growth rate is 28% in the European market between 2025–2033^4^). ICI-IA is currently quite a common problem in rheumatology clinics and will continue to increase.

ICI-IA results in functional impairment,^5^ which is often persistent, even after ICI discontinuation,^6^ and carries a large emotional and quality of life impact for patients.^7^ However, there is no high-quality body of evidence to guide treatment, with current recommendations not evidence based.^8^,^10^ Management may start with intra-articular glucocorticoid injections in mild cases, but when systemic immunosuppression is required, the current first-line therapy is glucocorticoids. Moderate glucocorticoid doses are inadequate for inducing drug or steroid-free remission in many patients with ICI-IA; however, higher doses (≥60 mg/day prednisolone) are associated with poorer oncological outcomes.^11^ Failure to control ICI-IA with glucocorticoids leads to the introduction of disease-modifying anti-rheumatic drugs (DMARDs), such as methotrexate or leflunomide, followed by biological DMARDs such as anti-tumour necrosis factor (TNF) or anti-interleukin-6 (IL-6) therapy. This treatment sequencing approach is associated with prolonged immunosuppression and may contribute to persistence,^6 12^ since data from other forms of inflammatory arthritis suggest a ‘window of opportunity’ in very early disease during which effective therapy results in milder disease or remission.^13 14^

TNF inhibition has shown efficacy in refractory ICI-IA,^2 15 16^ consistent with high TNF expression in synovial biopsy tissue.^17^ Anti-TNF agents are very well-tolerated with a good safety profile and proven efficacy in rheumatoid arthritis (RA) and psoriatic arthritis (PsA). Emerging data from ICI-IA synovial fluid show the presence of clonally expanded effector CD8+ T-cell populations that are highly productive of TNF^5^ and likely recruited by an activated myeloid population also involving TNF.^18^ Infiltrating CD8+ T-cell numbers within melanoma correlate with response to ICI but are reduced by TNF.^19^,^21^ Thus, TNF may provide a tumour escape mechanism in the context of ICI therapy. Indeed, in humanised mice, ICI treatment alone can induce inflammatory arthritis associated with TNF-producing T cells, against which anti-TNF therapy is efficacious.^22^ The ability of anti-TNF given in combination with ICI to improve ICI cancer outcomes has been tested in a phase 1b clinical trial and found no adverse safety signal.^23 24^ Together, these data make TNF an attractive target in ICI-IA.

Therefore, the REACT trial (REmission induction of Arthritis caused by Cancer ImmunoTherapy) has been designed in answer to recent calls for a paradigm shift^11 25 26^; it aims to test whether earlier use of more targeted therapies, specifically anti-TNF instead of glucocorticoids, will lead to more effective and rapid symptom control from ICI-IA, reduce glucocorticoid exposure, prevent early ICI discontinuation and increase the frequency of drug-free ICI-IA remission. Data on cancer outcomes will also be collected.

## Methods and analysis

### Patient and public involvement

A patient group with lived experience of ICI-IA helped shape the proposal that was submitted for funding through discussion of key questions. They contributed to the trial design (current standard of care vs anti-TNF alone) choosing this over other options including standard of care plus anti-TNF or placebo; the consideration to not over-immunosuppress patients was thought to be important for patient acceptability. Visit burden and acceptability of ultrasound-guided biopsy were also discussed.

A patient partner was a co-applicant during funding acquisition with both the trial management group (TMG) and trial steering committee (TSC) each having at least one patient partner as a member.

Patient partners were also involved in trial protocol finalisation, in particular, playing a key role in the development of a patient-centred composite outcome measure, itself used as an exploratory outcome for REACT (see Discussion). Details on the development of this will be published separately.

Patients have and will continue to review the patient information sheet and consent forms. Patient partners will also continue to contribute to newsletters, lay summary results and infographics and publicity about the trial; they will also help disseminate results of the trial via patient networks and public events.

### Trial design

**Figure 1:**
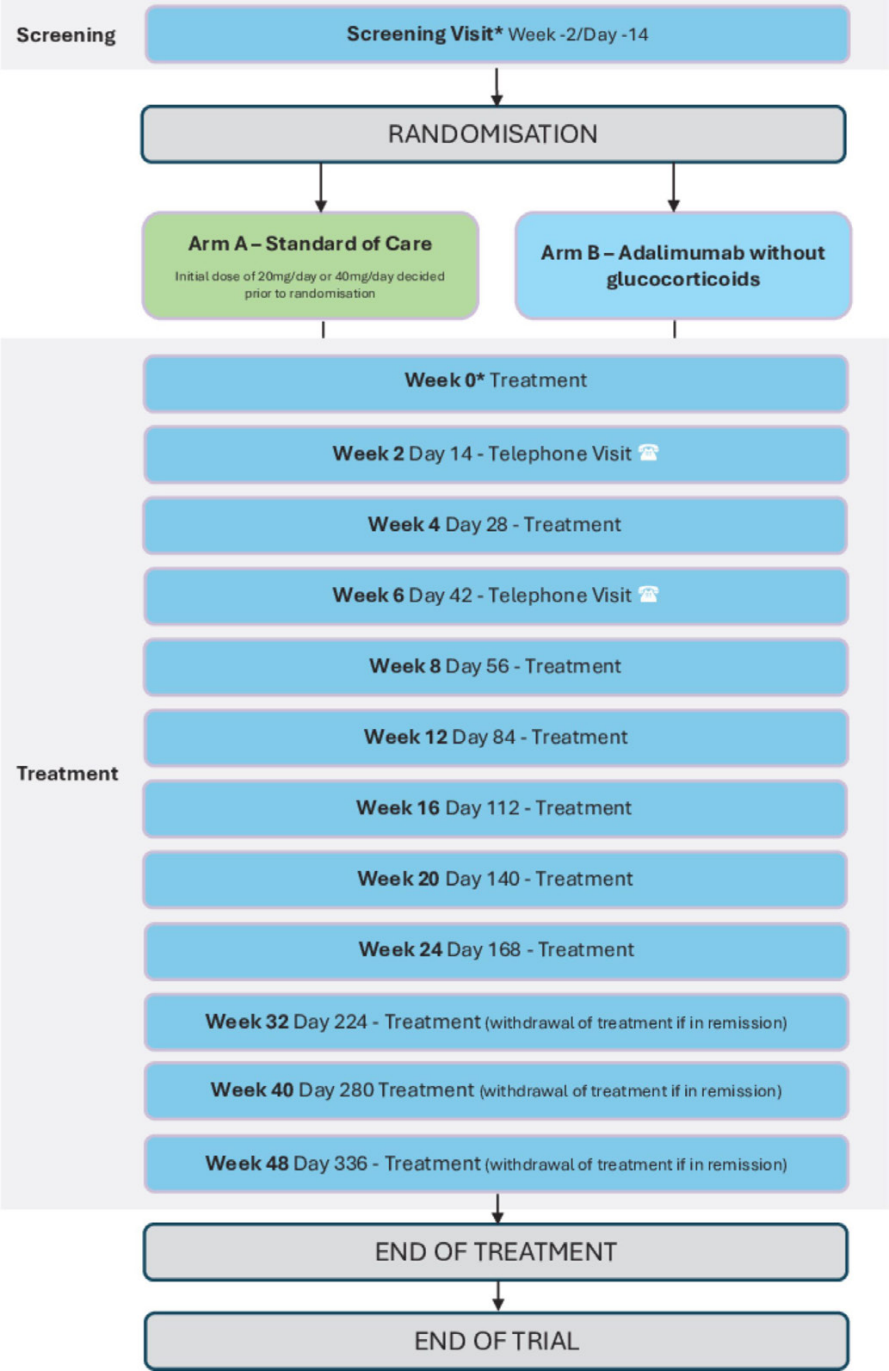
Schema for REACT trial. *Screening visit and Week 0 can be conducted on the same day. REACT, REmission induction of Arthritis caused by Cancer ImmunoTherapy.

REACT is a prospective, multicentre, open-label, superiority, two-arm, randomised controlled clinical trial to guide initial therapy for patients with ICI-IA. The trial will compare the current standard of care (prednisolone; Arm A) to adalimumab without glucocorticoids (Arm B) (figure 1). REACT aims to recruit 70 patients (35 in each arm) from >10 UK hospitals.

During the 48-week treatment period, participants have regular contact with the participating clinical and research teams either in person or via the telephone, according to the patient pathway (online supplemental appendix 1).

The WHO Trial Registration Data Set is included in online supplemental appendix 2.

The full protocol can be requested from the REACT Trial Office (REACT@trials.bham.ac.uk).

### Consent

Written informed consent is obtained by the investigator (or an appropriately trained and suitably delegated sub-investigator, who must be a Specialist Trainee level doctor or above). A patient information sheet is provided to facilitate this process (online supplemental appendix 3). If the patient agrees to participate, an informed consent form must be signed when the patient attends their first clinic appointment, prior to their entry onto the trial (online supplemental appendix 4).

### Randomisation

Participants are randomised 1:1 to receive either standard of care (prednisolone; Arm A) or adalimumab without glucocorticoids (Arm B) by the investigator or suitably trained delegate. Randomisation will be performed via a computer programme developed by the Cancer Research UK Clinical Trials Unit (CRCTU). The personnel randomising participants will not have access to the random allocation sequence. To avoid any possibility of the treatment allocation becoming too predictable, a 20% random element has been included in the randomisation algorithm. Stratification variables are tumour type (melanoma or non-melanoma); ICI agents used (single or combination); planned initial prednisolone dose (20 mg or 40 mg); duration of symptoms (≤12 weeks or >12 weeks); and participation in the optional biopsy sub-study.

### Patient selection

Patients are identified through clinics, multi-disciplinary teams and the acute oncology service. Oncologists identifying a potential participant will refer for rheumatology assessment at a participating hospital who will then consent the patient prior to confirming eligibility and subsequent randomisation.

Box 1 contains the key eligibility criteria.

Box 1 **Eligibility criteria for the REACT trial**

**Inclusion criteria**

- Informed consent must be obtained prior to any trial-related procedures being performed.
- Able to understand and comply with the requirements of the trial.
- Inflammatory arthritis with at least one clinically swollen joint at screening.
- Male and female participants aged ≥18 years.
- Patient treated with immune checkpoint inhibitor (ICI) concurrent with or last dose within 12 weeks prior to the onset of inflammatory arthritis.
- Decision of two treating physicians, one of whom must have expertise in the management of inflammatory arthritis, that treatment with systemic glucocorticoids would be appropriate management for their inflammatory arthritis. Details of the physicians who have decided this will be documented in the source data.

**Exclusion criteria**

- If currently using oral glucocorticoids, use must not exceed 2 weeks prior to baseline (except hydrocortisone for adrenal replacement where longer term use is allowed).
- Pre-existing (prior to first use of ICI) inflammatory arthritis due to rheumatic autoimmune disease.
- The arthritis not considered, by the treating physician, to be immune checkpoint inhibitor-induced inflammatory arthritis (ICI-IA).
- Active or latent tuberculosis (TB) (unless having fulfilled chemoprophylaxis management according to local guidelines) or other chronic infection considered a contraindication to anti-TNF.
- Positive test for HIV, Hepatitis C (HCV) (evidenced by both positive anti-HCV antibody and HCV-RNA in serum), Hepatitis B Virus (HBV) as evidenced by positive hepatitis B surface antigen (HBsAg) or anti-hepatitis B core antibodies (HBcAb). Participants with a negative result in the last 6 months will be eligible.
- History of demyelinating disorder.
- Hypersensitivity to the active substance or to any of the excipients in the adalimumab preparation.
- Any medical, surgical or psychiatric condition that the investigator believes may jeopardise the participant, or the validity of the trial results, were they to participate in the trial.
- Moderate to severe heart failure (New York Heart Association III/IV).
- Concurrent use of other biological immunosuppressive drugs or Janus kinase inhibitors.
- Live vaccine received less than 4 weeks before first dose of trial drug.
- Any current severe infection.
- Known ocular herpes simplex.

REACT, REmission induction of Arthritis caused by Cancer ImmunoTherapy.

### Interventions and dose modifications

#### Arm A—standard of care

The comparator will follow usual care for this patient population. A reducing course of oral prednisolone with a starting dose of 20 mg/day or 40 mg/day decided prior to randomisation on clinical grounds by the treating rheumatologist.

Guidance on tapering and management in case of non-response or relapse on prednisolone withdrawal is provided in online supplemental appendix 5.

If arthritis fails to remit, further therapy may be provided according to clinical practice and American Society of Clinical Oncology (ASCO) recommendations.^8^ This may include further oral prednisolone with or without oral DMARDs such as methotrexate, leflunomide or hydroxychloroquine, titrating up to a biological DMARD (anti-TNF or anti-IL6R) if required. We recommend avoiding sulfasalazine given the experience of hypersensitivity reactions in this context.^10 27 28^ With disease control, therapy will be titrated down to the minimum amount required to control synovitis if drug cessation is not possible.

#### Arm B—adalimumab without glucocorticoids

Adalimumab 40 mg every other week by subcutaneous injection. Participants will be taught how to self-administer by the trial investigators.

Investigators may stop adalimumab at Week 24 or after, provided there has been evidence of sustained remission defined as the absence of synovitis on clinical examination on at least two consecutive occasions 4 weeks apart. If synovitis recurs following this, then adalimumab can be restarted. With inadequate response at Week 12 or after, the dose of adalimumab can be increased to 80 mg every other week (online supplemental appendix 5).

If arthritis fails to remit, then further therapy may be required in addition to adalimumab, or in place of adalimumab if the participant does not respond. Further therapy may include oral prednisolone, oral DMARDs or an alternative biological DMARD. With disease control, therapy will be titrated down to the minimum amount required to suppress synovitis if drug cessation is not possible, although adalimumab will not be stopped before Week 24 except in the event of non-response or toxicity.

### Treatment compliance

Drug accountability logs are not required for prednisolone. The local trial pharmacist is responsible for maintaining and updating drug accountability logs for adalimumab to monitor compliance. In addition, participants will be issued with a diary to complete each day, recording the dosage of medication and whether any doses were missed. The diary also includes a section where participants can record any relevant information such as side effects suffered or reasons for missed doses. The completed diary will be collected by the site at each protocol scheduled visit and checked.

### Concomitant medication

#### Arm A—standard of care

Use of biological DMARDs such as TNF or IL-6R inhibitors is prohibited in the first 12 weeks after baseline and their use within this period would result in the participant being considered a non-responder.

#### Arm B—adalimumab without glucocorticoids

If taking glucocorticoids at screening (other than for adrenal insufficiency), use must not exceed 2 weeks prior to the baseline visit with an aim to discontinue use at baseline on first administration of adalimumab. However, further glucocorticoids may be used in this arm if clinically indicated. Use of other biological DMARDs (eg, anakinra and abatacept) or other TNF-antagonists is not recommended concomitant with adalimumab based on the possible increased risk for infections, including serious infections and other potential pharmacological interactions.

#### Both arms

Co-treatment with Cytochrome P450, family 3, subfamily A (CYP3A) inhibitors, including cobicistat-containing products, is expected to increase the risk of systemic side-effects with prednisolone and should be monitored and managed as per standard practice by the investigator.

Live vaccines should be avoided while the participant is on active treatment within the trial.

### Trial outcomes

The primary outcome is glucocorticoid-free arthritis remission rate at 24 weeks where remission is defined as: (i) No use of systemic or intra-articular glucocorticoids (except when used for adrenal insufficiency) within 4 weeks prior to assessment at 24 weeks; and (ii) absence of synovitis on clinical examination.

Key secondary outcomes include time to arthritis remission, drug-free arthritis remission at 48 weeks, 66/68 swollen and tender joint counts, patient acceptable symptom state, cumulative glucocorticoid exposure, other IrAEs, health-related quality of life, cancer response and overall survival. The full list of secondary and exploratory outcomes is listed in online supplemental appendix 6.

Where an Investigator removes a participant from the trial or if the participant declines further participation, final Week 24 or Week 48 assessments will still be performed, if possible, as long as consent is not withdrawn. If a participant ceases trial treatment due to adverse clinical or laboratory events, they will be treated and monitored according to accepted medical practice and continue to attend the remaining trial visits if they agree.

### Statistical analysis plan

A formal statistical analysis plan (SAP) for REACT provides a detailed description of the planned analyses and can be requested from the REACT Trial Office (REACT@trials.bham.ac.uk). Unless reported otherwise, the threshold for determining statistical significance from hypothesis testing is p<0.05. Where a parametric and non-parametric test has been specified dependent on the normality of data distribution, this will be assessed using a q-q plot, or a Shapiro-Wilk test where p<0.05 will be considered to indicate data is non-normally distributed. A brief outline of planned analyses has been included.

#### Outcome analyses

The safety population will include all patients who receive any trial treatment.

The modified intention-to-treat population for the primary analyses will include all patients who receive any trial treatment and who have a baseline joint assessment measurement and at least one further joint assessment post-baseline. No imputation of the primary outcome is planned for patients missing a 24-week assessment; instead, the trial sample size has been inflated to account for patient withdrawal. For the secondary endpoints, this includes all patients who receive any trial treatment and have available data for the respective outcome measure.

The intention-to-treat population includes all randomised patients in their treatment arms who have available data for the respective outcome measure.

Glucocorticoid-free arthritis remission rate at 24 weeks will be calculated for each treatment arm by dividing the number of patients reaching the response criteria by the total number of patients randomised to each arm. Failures in glucocorticoid-free arthritis remission recorded prior to the 24-week outcome (eg, due to early initiation of biological DMARD trial) will be used in this assessment. Where patients are missing the primary outcome (eg, because their Week 24 assessment is missing), they shall be treated as a failure and included in the denominator only. In addition, the number of such patients in each arm will be reported. 95% CIs will be calculated for each rate.

The remission rate will be compared between arms using a χ^2^ or Fisher’s exact test. Moreover, we will use logistic regression models, adjusted for stratification variables and other baseline covariates deemed to be associated with the outcome (such as patient standard of care). This will allow us to present the treatment effect for each of the stratification variables, as well as attempt to distinguish efficacy effects relating to standard of care compared with those because of trial treatment.

Analyses of secondary and exploratory measures are included in online supplemental appendix 7.

#### Sample size calculation

Sample size calculations were performed with a power of 80% and a significance level of 5%, with a treatment difference in remission rate of at least 34%; 21% remission rate in standard of care compared with 55% in the anti-TNF treatment arm. The calculated sample size was determined to be 62 patients, with a 10% dropout allowance increasing the total trial size to 70 patients when rounded and split evenly between arms.

The Birmingham ICI-IA cohort was used to identify glucocorticoid-free remission rate in the control arm. Remission rates on treatment in inflammatory arthritis with anti-TNF vary by disease and study design. Remission rates in established RA of 47% are seen at 6 months. Factors associated with drug-free remission are absence of anti-CCP antibodies, male sex and shorter RA symptoms, all of which favour a higher remission rate in ICI-IA when compared with a RA population. ICI-IA shows greater clinical resemblance to PsA than RA and real-world remission rates in PsA are 60% and up to 81% in early PsA. Therefore, the remission rate in the anti-TNF arm is a conservative estimate.

#### Subgroup and interim analyses

The main estimators will be adjusted for the stratification variables (see Randomisation section), with results presented for each stratum; no further subgroup analyses are planned. There are no planned interim analyses.

No formal stopping rules have been established; however, on recommendation of the independent data monitoring committee (DMC), trial stoppage may occur dependent on the results of an ad hoc interim analysis.

### Harms reporting and analysis

Adverse events (AEs) are measured by National Cancer Institute (NCI) Common Terminology Criteria for Adverse Events, V.5.0.^29^ Definitions of different types of AEs are listed in online supplemental appendix 8. The reporting period for AEs will be between the date starting trial treatment (Week 0) until Week 48.

Side effects are commonly encountered in patients receiving adalimumab, prednisolone or other anti-arthritic drugs that may subsequently be used in either arm of REACT. As the safety profiles of the drugs used in this trial are well-characterised, only adverse reactions (ARs), those harms that are related to the arthritis interventions, will be reported together with new IrAEs. All ARs will be reported using electronic case report forms (eCRFs). However, all AEs that meet the definition of a serious adverse event (SAE; see online supplemental appendix 8) even if they are unrelated to the arthritis interventions will be reported.

Respiratory and systemic non-opportunistic infections are very common and are recognised ARs with adalimumab and glucocorticoids. Hospitalisations for these events may be completed on an expected serious adverse reaction form and do not require expedited reporting.

Any death occurring during the protocol defined follow-up period, whether considered related or not, must be reported as an SAE within 24 hours of the investigator becoming aware of the event.

### Tissue and blood samples

A sub-study will collect serum and whole blood at screening and Week 12, as well as optional ultrasound-guided synovial tissue biopsies at selected centres pre- (Week 0) and post-intervention (Week 12). Consent for participants is optional for synovial biopsy and baseline DNA collection.

Biopsy samples in formalin will be shipped to the University of Birmingham for paraffin embedding and storage. Blood samples will be processed for sera at site and stored frozen together with whole blood for DNA and RNA and whole blood in Cytodelics mix before shipment in batches to the University of Birmingham.

All samples will be collected in accordance with national regulations and requirements including standard operating procedures for logistics and infrastructure. Samples will be taken in appropriately licensed premises, stored and transported in accordance with the Human Tissue Authority guidelines and National Health Service (NHS) trust policies.

### Data management

Electronic CRFs are being used and completed online. Authorised staff at hospital sites will require an individual secure login username and password to access this online data entry system. Any paper CRFs must be completed, signed/dated and returned to the REACT Trial Office by the investigator or an authorised member of the site research team. Data reported on each CRF should be consistent with the source data or the discrepancies should be explained. If information is not known, this must be clearly indicated on the CRF. All missing and ambiguous data will be queried. All sections are to be completed.

All trial records must be archived and securely retained for at least 20 years. No documents will be destroyed without prior approval from the sponsor, via the central REACT Trial Office.

On-site monitoring will be carried out as required following a risk assessment and as documented in the Quality Management Plan. Any monitoring activities will be reported to the central REACT Trial Office and any issues noted will be followed up to resolution. REACT will also be centrally monitored, which may trigger additional on-site monitoring.

### Data and sample sharing

The CRCTU will hold the final trial dataset and will be responsible for the controlled sharing of anonymised clinical trial data with the wider research community to maximise potential patient benefit while protecting the privacy and confidentiality of trial participants. Data anonymised in compliance with the Information Commissioner’s Office requirements, using a procedure based on guidelines from the Medical Research Council Methodology Hubs, will be available for sharing with researchers outside of the trials team within 6 months of the primary publication.

### Trial organisation structure

The University of Birmingham is acting as sponsor for this multicentre study: Research Ethics, Governance & Integrity, Research Strategy and Services Division, Research Park, Birmingham, B15 2TT. Email: researchgovernance@contacts.bham.ac.uk). The trial is being conducted under the auspices of the CRCTU, University of Birmingham, according to their local procedures.

The TMG is responsible for the day-to-day running and management of the trial. Members of the TMG include the Chief Investigator, Co-Investigators (which includes a patient partner), the Trial Management Team Leader (or delegate), the Trial Biostatistician, the Trial Coordinator and the Monitor. The TMG will meet every month during recruitment and at least yearly thereafter.

An independent TSC supervises the conduct of the trial, monitoring progress including recruitment, data completeness, losses to follow-up and deviations from the protocol. Membership includes independent clinicians (including at least one oncologist and one rheumatologist), an independent statistician and at least one patient partner. The TSC meets at least annually after the DMC meeting. The TMG will report to the TSC.

Data analyses will be supplied in confidence to the independent DMC, which will be asked to give advice on whether the accumulated data from the trial, together with the results from other relevant research, justifies the continuing recruitment of further participants. Membership includes independent clinicians (including at least one oncologist and one rheumatologist), and an independent statistician. The DMC meets at least annually while participants are on treatment. Additional meetings may be called if recruitment is much faster than anticipated and the DMC may, at their discretion, request to meet more frequently. An emergency meeting may also be convened if a safety issue is identified. The DMC will report to the TMG, who will convey the findings of the DMC to TSC, Medicines for Healthcare products Regulatory Agency, funders and/or sponsors as applicable. The DMC may consider recommending the discontinuation of the trial if the recruitment rate or data quality is unacceptable or if any issues are identified which may compromise participant safety. The trial would also stop early if the interim analyses showed differences between treatments that were deemed to be convincing to the clinical community.

All groups and committees operate in accordance with a trial-specific charter based on the template created by the Damocles Group.

### Confidentiality

Confidential information collected during the trial will be stored in accordance with the General Data Protection Regulation 2018. As specified in the patient information sheet and with the patients’ consent, participants will be identified using only their date of birth and unique trial ID number. Authorised staff may have access to the records for quality assurance and audit purposes. The Trials Office maintains the confidentiality of all participants’ data and will not disclose information by which participants may be identified to any third party other than those directly involved in the treatment of the participant and organisations for which the participant has given explicit consent for data transfer (eg, laboratory staff).

### Trial status

Recruitment for the trial opened in Feb-2025 and is scheduled to last until September 2026.

### Ethics and dissemination

The trial will be performed in accordance with the recommendations guiding physicians in biomedical research involving human subjects, adopted by the 18th World Medical Association General Assembly, Helsinki, Finland and stated in the respective participating countries’ laws governing human research, and Good Clinical Practice. The current protocol (V.2.0 dated 18 October 2024) was approved on 31 October 2024 by East Midlands—Leicester South Research Ethics Committee (Ref: 24/EM/0202). Participants are required to provide written informed consent. The REACT Trial Office will coordinate and communicate protocol modifications and modifications to all relevant parties.

A meeting will be held after the end of the study to allow discussion of the main results among the collaborators prior to publication. The results of this trial will be disseminated through national and international presentations and peer-reviewed publications. A lay summary of the results will be published online and provided to trial sites to share with patients.

Manuscripts will be prepared by the TMG and authorship will be determined by mutual agreement. Any secondary publications and presentations prepared by Investigators must be reviewed by the TMG.

## Discussion

### Strengths of the REACT trial

Adalimumab is currently the most widely used anti-TNF agent in the UK, which is one of the main strengths of the REACT trial. Alongside the availability of biosimilars, the convenience of subcutaneous over intravenous administration will also reduce cost and potentially increase patient acceptability. An alternative anti-TNF that is available in biosimilar format is etanercept. However, this is a construct based on recombinant TNF receptors that will also inhibit the effect of lymphotoxin in addition to TNFα; whether there are additional safety implications of lymphotoxin inhibition in this context is unknown. Although a retrospective analysis of anti-TNF use in ICI-IA showed a faster time to arthritis control, a shorter time to cancer progression was also suggested.^30^ However, the inherent biases of non-randomised, retrospective studies undermine their informativeness for clinical practice, and notably analyses were not adjusted for glucocorticoid doses that were higher in the anti-TNF treated arthritis group, emphasising the need for randomised trials such as REACT.

REACT’s design compares current standard of care beginning with glucocorticoid use, as reflected in widespread clinical practice and guidelines, to adalimumab without glucocorticoids. This was seen as a strength by the patient partners as they were keen that the trial should seek to control ICI-IA in each arm and to avoid unnecessary over-immunosuppression and use of placebos. Furthermore, this was an important consideration for oncologist stakeholders who wanted to investigate treatments that minimised glucocorticoid exposure.

The lack of placebo in this pragmatic strategy trial is also seen as a strength as it reduces burden, complexity and cost of the trial:

- Placebo use would have been necessary in each arm ie, one arm would have placebo anti-TNF, and the other placebo glucocorticoids. This would increase patient burden, particularly if subsequent therapies need to be added and considering the background cancer diagnosis.
- Placebo glucocorticoid would pose particular problems as this would need to be titrated down in accordance with usual clinical practice with a view to stopping. In our experience, it is relatively common for a patient who was initially doing well to flare on glucocorticoid reduction and therefore this would lead to a high rate of unblinding. Further, if the 20 mg dose of prednisolone is chosen and there is no initial response, a common clinical strategy would be to escalate to 40 mg.
- In the anti-TNF arm, if a patient responded but still had low activity, then consideration could be given to adding methotrexate or hydroxychloroquine, alongside the anti-TNF injection and the placebo glucocorticoid. If the patient only partially responded or non-responded, then glucocorticoid therapy may be considered clinically appropriate. However, not knowing which arm the patient was in would necessitate unblinding.
- Were the patient to develop another IrAE requiring systemic treatment during the trial, this would also necessitate unblinding, since glucocorticoids are typically first-line management.

Another major strength of the trial is the development of a specific patient-centred ranked composite outcome, co-produced with our patient partners, increasing the relevance of the trial results to the patient population. REACT represents one of the first trials in ICI-IA; therefore, how patients and clinicians would prioritise oncological, rheumatological and quality of life outcomes is currently unknown. The aim is to incorporate a patient’s overall status and treatment response and allow patients to inform the analyses performed. The ranking will be via consensus among clinicians and patients, informed by a literature review of all published trials and studies in ICI-IA and clinical trials of ICI, a focus group incorporating patient partners, rheumatologists and oncologists and a survey. Ultimately, this will allow us to retrospectively rank all patients according to the desirability of their overall status and compare their position between treatment arms.

Finally, a key asset of REACT is the optional biopsy sub-study, which will generate a unique, highly valuable bioresource, allowing for comparisons of treatment effects on ICI-IA tissue.

### Weaknesses of the REACT trial

Although we have highlighted above the many strengths of the non-placebo-controlled approach within the REACT trial, the use of placebo is still deemed by many as the gold standard on which to determine efficacy and provide clear evidence of effectiveness (or the lack of it) when testing a new treatment. Therefore, this must be considered a limitation to the trial’s design.

When REACT was being designed, inhibition of TNF was determined to be the best approach for a pragmatic strategy ICI-IA trial. Since then, data suggest that IL-6 inhibition may have a similar efficacy for ICI-IA and, like anti-TNF, may also show synergistic effects with ICI in animal models of cancer.^31^ Incorporation of this question could, therefore, be perceived as a missed opportunity within REACT. However, at that time, there was less clinical experience with tocilizumab in this clinical setting, availability was restricted due to use in COVID-19, and there were no biosimilars with significantly higher drug costs compared with adalimumab.

Although loading doses of adalimumab are not typically used in RA, they lead to less frequent primary non-response and increased longevity of response in inflammatory bowel disease.^32 33^ Indeed, pharmacokinetic-pharmacodynamic modelling in RA suggests a 160 mg loading dose will reduce time to efficacy.^34^ Therefore, not using them within REACT is a potential weakness as steady state drug levels will be reached more slowly and synovial inflammation controlled less quickly. While the possibility of their use was explored, during funder review, it was deemed that the evidence from inflammatory bowel disease for the use of 160 mg then 80 mg loading doses was insufficient to justify their use in this context and so a dose of 40 mg every 2 weeks without loading was selected.

### Summary

ICI-IA is a new clinical entity with limited high-quality evidence with which to guide treatment. It is complicated by the presence of an underlying cancer, which the ICI-mediated immune stimulation is seeking to treat. Glucocorticoids are typically used as first-line therapy, but data suggest that high peak doses of glucocorticoids are associated with poorer survival.^11 35^ Poorly controlled ICI-IA, or prednisolone doses>10 mg, may lead to ICI being withheld.^8^ Furthermore, persistence of ICI-IA is common even after ICI cessation. Therefore, we aim to test whether early use of effective, targeted therapy may lead to faster ICI-IA control, less glucocorticoid use and interruption of ICI therapy, and ultimately lead to higher rates of drug-free arthritis remission within the REACT trial.

## Supplementary material

10.1136/bmjopen-2026-116847 online supplemental file 1
