# Supplementary material for: Pragmatic, open-label, multicentre, randomised controlled trial to guide initial therapy for immune checkpoint inhibitor-induced inflammatory arthritis comparing standard of care (prednisolone) to adalimumab without glucocorticoids: REACT trial protocol
Source: BMJ Open. 2026 Mar 3;16(3):e116847. doi: 10.1136/bmjopen-2026-116847 (PMC12958871; doi:10.1136/bmjopen-2026-116847)
Supplement: online supplemental file 1 [file bmjopen-16-3-s001.pdf]

Appendix 1. Patient pathway through the REACT trial

**Visit Name**	**Screening**	**Week 0**	**Week 2**	**Week 4**	**Week 6**	**Week 8**	**Week 12**	**Week 16**	**Week 20**	**Week 24**	**Week 32**	**Week 40**	**Week 48**
**Week**	**Week -2**	**Week 0**	**Week 2 ☎**	**Week 4**	**Week 6 ☎**	**Week 8**	**Week 12**	**Week 16**	**Week 20**	**Week 24**	**Week 32**	**Week 40**	**Week 48 EOT**
Day	-14[1]	0	14	28	42	56	84	112	140	168	224	280	336
Telephone consult			Yes[2]		Yes								
Visit Window (days)			± 3	± 3	± 3	± 3	± 5	± 5	± 5	± 5	± 7	± 7	± 7
Informed Consent	X												
Eligibility Assessment	X												
Randomisation	X[3]												
Demography	X												
Medical History	X												
Chest X-ray[4]	X												
Prednisolone initial prescription decision[5]	X												
Hepatitis and HIV screen[6]	X												
TB Test[7]	X												
ANA, Rheumatoid factor, anti-CCP[8]	X												
Pregnancy test[9]	X		(X)	(X)	(X)	(X)	(X)	(X)	(X)	(X)	(X)	(X)	(X)
Height/weight	X												
Physical Examination[10]	X	X		X		X	X	X	X	X	X	X	X
Vital signs (BP/pulse/ temperature)	X	X		X		X	X	X	X	X	X	X	X
Routine bloods[11]	(X)	X		(X)		(X)	X	(X)	(X)	(X)	(X)	(X)	(X)
CRP	X	X		X		X	X	X	X	X	X	X	X

**Visit Name**	**Screening**	**Week 0**	**Week 2**	**Week 4**	**Week 6**	**Week 8**	**Week 12**	**Week 16**	**Week 20**	**Week 24**	**Week 32**	**Week 40**	**Week 48**
**Week**	**Week -2**	**Week 0**	**Week 2 ☎**	**Week 4**	**Week 6 ☎**	**Week 8**	**Week 12**	**Week 16**	**Week 20**	**Week 24**	**Week 32**	**Week 40**	**Week 48 EOT**
Day	-14[1]	0	14	28	42	56	84	112	140	168	224	280	336
Telephone consult			Yes[2]		Yes								
Visit Window (days)			± 3	± 3	± 3	± 3	± 5	± 5	± 5	± 5	± 7	± 7	± 7
66/68 Swollen and tender joint counts	X	X		X		X	X	X	X	X	X	X	X
Medication Review[12]	X	X	X	X	X	X	X	X	X	X	X	X	X
Adverse/Clinical Events		X	X	X	X	X	X	X	X	X	X	X	X
Adalimumab every 2 weeks **OR** standard of care until remission		X	X	X	X	X	X	X	X	X	X[13]	X[13]	X[13]
Investigator-assessed Cancer Status[14]		X		X		X	X	X	X	X	X	X	X
Physician Global Arthritis VAS		X		X		X	X	X	X	X	X	X	X
Patient Global Disease Activity Assessment VAS		X		X		X	X	X	X	X	X	X	X
Pain VAS		X		X		X	X	X	X	X	X	X	X
Fatigue VAS		X		X		X	X	X	X	X	X	X	X
Patient Acceptable Symptom State (PASS)		X		X		X	X	X	X	X	X	X	X
HAQ-DI		X					X			X			X
EQ-5D-5L		X					X			X			X
ICECAP-A		X					X			X			X

Visit Name	Screening	Week 0	Week 2	Week 4	Week 6	Week 8	Week 12	Week 16	Week 20	Week 24	Week 32	Week 40	Week 48
**Week**	**Week -2**	**Week 0**	**Week 2 ☎**	**Week 4**	**Week 6 ☎**	**Week 8**	**Week 12**	**Week 16**	**Week 20**	**Week 24**	**Week 32**	**Week 40**	**Week 48 EOT**
Day	-14[1]	0	14	28	42	56	84	112	140	168	224	280	336
Telephone consult			Yes[2]		Yes								
Visit Window (days)			± 3	± 3	± 3	± 3	± 5	± 5	± 5	± 5	± 7	± 7	± 7
Blood for research (serum and whole blood)		X					X						
Optional synovial biopsy[15]		X					X						
Independent joint count assessment										X			X
RECIST 1.1[16]										X			X

ANA, antinuclear antibody; BP, blood pressure; CCP, cyclic citrullinated peptide; CRP, c-reactive protein; EOT, end of trial; HIV, human immunodeficiency virus; RECIST, response evaluation criteria in solid tumors; TB, tuberculosis; VAS, visual analogue scale.

☎ - telephone visit, not in person.

[1] Screening visit and Week 0 can be conducted on the same day if all results necessary for screening are available. Ideally, baseline dosing will begin as soon after screening as possible, although a 14-day maximum screening window is allowed.

[2] Participants in adalimumab arm may attend for injection if further training required for self-injection.

[3] Randomisation only after all eligibility has been confirmed.

[4] Only if no chest imaging within the previous 6 months.

[5] Treatment decision made by rheumatologist.

[6] Hep B surface antigen and anti-HepB sAb and cAb, Hepatitis C antibody and HIV at screening, if not already performed within the 6 months prior to screening.

[7] TB interferon-gamma release assay (IGRA) at screening if not already performed within the 6 months prior to screening.

[8] ANA, rheumatoid factor and anti-CCP at screening if not previously tested since inflammatory arthritis onset.

[9] For women of child-bearing potential, a (non-exclusionary) urine pregnancy test must be conducted at screening to ensure that the participant can make a fully informed decision about participation, and at the participant's first post-treatment trial visit to ensure that any pregnancy occurring during the treatment period is detected.

[10] Symptom-directed only.

[11] Full blood count, urea and electrolytes, liver function tests, plus glucose if on glucocorticoids. Routine bloods should be performed at Week 0 and Week 12. Routine bloods should be requested at screening if results from the previous 4 weeks not available. At all other visits consider obtaining bloods if results are not available from the interval between the last scheduled in-person assessment and if clinically indicated.

[12] Medications for disease control of inflammatory arthritis, other immune-related adverse events, cancer, serious adverse event medication, and immunosuppression for any other indication ONLY. The Patient Diary should be given to the patient at Week 0 and reviewed at subsequent visits.

[13] Withdrawal of treatment if in remission.

[14] Based on last available oncology assessment.

[15] Week 0 biopsy can take place up to 14 days prior to the visit date. Week 0 research bloods and optional biopsy must be taken prior to trial drug administration (adalimumab or standard of care), expect where glucocorticoids have already been administered prior to screening.

[16] Based on most recent existing imaging done as part of clinical care.

Appendix 2. World Health Organization trial registration data set

Data category	Information
Primary registry and trial identifying number	ISRCTN: 18217497
Date of registration in primary registry	28-Nov-2024
Secondary identifying numbers	n/a
Source(s) of monetary or material support	NIHR/MRC Efficacy and Mechanism Evaluation (EME) Programme (reference: NIHR154840)
Primary sponsor	University of Birmingham
Secondary sponsor(s)	n/a
Contact for public queries	REACT@trials.bham.ac.uk
Contact for scientific queries	b.fisher@bham.ac.uk
Public title	A trial to compare current standard treatment of Immune Checkpoint Inhibitor-induced Inflammatory Arthritis (ICI-IA), with treatment using adalimumab without glucocorticoids: REACT
Scientific title	REmission induction of Arthritis caused by Cancer ImmunoTherapy (REACT): a randomised, multicentre trial to guide initial therapy for immune checkpoint inhibitor-induced inflammatory arthritis
Countries of recruitment	UK
Health condition(s) or problem(s) studied	Immune checkpoint inhibitor-induced inflammatory arthritis
Intervention(s)	Arm A: Standard of care (prednisolone)<br>Arm B: Adalimumab without glucocorticoids
Key inclusion and exclusion criteria:	Ages eligible for study: Aged 18 and over<br>Sexes eligible for study: Both<br>Accepts healthy volunteers: No

Data category	Information
	Inclusion criteria: Inflammatory arthritis with at least one clinically swollen joint at screening; patient treated with immune checkpoint inhibitors; able to provide written informed consent
	Exclusion criteria: If currently using oral glucocorticoids, use must not exceed 2 weeks prior to baseline; pre-existing (prior to first use of immune checkpoint inhitiors) inflammatory arthritis due to rheumatic autoimmune disease; active or latent tuberculosis
Study type	Interventional
	Allocation: Two-arm, randomised, open-label trial
	Primary purpose: Efficacy
	Phase III
Date of first enrolment	03-Apr-2025
Target sample size	70 (randomised 1:1)
Recruitment status	Open
Primary outcome(s)	To determine the proportion of patients in glucocorticoid-free remission 24 weeks from initiation of anti-tumour necrosis factor (TNF) therapy compared to initial glucocorticoids use alone.
Secondary outcome(s)	1. Time to remission defined as time from randomisation to first absence of synovitis on clinical examination at Weeks 0, 4, 8, 12, 16, 20, 24, 32, 40, 48<br>2. Arthritis remission measured as absence of synovitis at 24 and 48 weeks<br>3. Drug-free arthritis remission measured as absence of synovitis with no immune checkpoint inhibitor-induced inflammatory arthritis (ICI-IA) treatment in the previous 4 weeks at 48 weeks<br>4. 66/68 swollen and tender joint counts, defined as the number of joints that are swollen and the number of joints that are tender at Screening, Weeks 0, 4, 8, 12, 16, 20, 24, 32, 40, 48<br>5. Patient arthritis disease activity global visual analogue scale (VAS) (0-100), measured in millimetres along a 10cm line on which participants mark to rate their perceived health in the past week, at Weeks 0, 4, 8, 12, 16, 20, 24, 32, 40, 48<br>6. Physician arthritis disease activity global VAS (0-100) , measured in millimetres along a 10cm line on which clinicians

Data category	Information
	mark to rate the participants' health in the past week, at Weeks 0, 4, 8, 12, 16, 20, 24, 32, 40, 48<br>7. Fatigue VAS (0-100), measured in millimetres along a 10cm line on which participants mark to rate their average fatigue in the past week, at Weeks 0, 4, 8, 12, 16, 20, 24, 32, 40, 48<br>8. Pain VAS (0-100), measured in millimetres along a 10cm line on which participants mark to rate their average pain in the past week, at Weeks 0, 4, 8, 12, 16, 20, 24, 32, 40, 48<br>9. Cumulative exposure to glucocorticoids over 24 and 48 weeks, where a participants' cumulative dose of glucocorticoids will be calculated from their treatment start to 24 weeks, and from treatment start to 48 weeks<br>10. Cumulative exposure to anti-tumour necrosis factor (TNF) (or other biological Disease-Modifying Anti-Rheumatic Drug (DMARD) or targeted synthetic DMARD), where a participants' cumulative exposure to anti-TNF or targeted synthetic DMARD (including JAK inhibitors) treatment will be calculated from their treatment start to 24 weeks, and from treatment start to 48 weeks over 24 and 48 weeks<br>11. Cumulative exposure to other conventional synthetic DMARD over 24 and 48 weeks, where a participants' cumulative exposure to synthetic DMARD not included above will be calculated from their treatment start to 24 weeks, and from treatment start to 48 weeks<br>12. Cumulative exposure to Immune Checkpoint Inhibitor (ICI) over 24 and 48 weeks, where a participants' cumulative exposure to any ICI will be calculated from their treatment start to 24 weeks, and from treatment start to 48 weeks<br>13. A summary of adverse events related to arthritis interventions using Common Terminology Criteria for Adverse Events (CTCAE) version 5.0 at Weeks 0, 4, 8, 12, 16, 20, 24, 32, 40, 48<br>14. A summary of serious adverse events using CTCAE version 5.0 at Weeks 0, 4, 8, 12, 16, 20, 24, 32, 40, 48<br>15. A summary of other immune-related adverse events (number of organs and toxicity level) using CTCAE version 5.0 at Weeks 0, 4, 8, 12, 16, 20, 24, 32, 40, 48<br>16. A summary of functional status as assessed by the Health Assessment Questionnaire Disability Index (HAQ-DI) at Weeks 0, 12, 24, 48<br>17. A summary of health-related quality of life as assessed by EQ-5D-5L questionnaire at Weeks 0, 12, 24, 48<br>18. A summary of well-being as assessed by ICECAP-A questionnaire at Weeks 0, 12, 24, 48

Data category	Information
	19. A summary of investigator-assessed cancer status as complete response, partial response, stable disease, or disease progression at Weeks 0, 4, 8, 12, 16, 20, 24, 32, 40, 48<br>20. Cancer response at Weeks 24 and 48 as defined by Response Evaluation Criteria in Solid Tumors (RECIST) version 1.1<br>21. Overall survival as time to death defined as the length time from participant randomisation to death, at Weeks 0, 4, 8, 12, 16, 20, 24, 32, 40, 48<br>22. Progression-free survival of cancer as time to progression (defined as first occurrence of disease progression via cancer response using RECIST version 1.1 or investigator-assessed cancer status), or death at Weeks 0, 4, 8, 12, 16, 20, 24, 32, 40, 48

Appendix 3. Exemplar patient information sheet

<To be printed on local hospital headed paper>

REACT

Treating joints affected by Arthritis following Immunotherapy

Patient Information Sheet

WE INVITE YOU TO TAKE PART IN A RESEARCH TRIAL

- We would like to invite you to take part in a clinical trial
- Before you decide whether to take part, it is important for you to understand why the research is being done and what it will involve
- Please take time to read the following information carefully. Discuss it with friends and relatives if you wish
- You are free to decide whether or not to take part in this trial. If you choose not to take part, this will not affect the care you get from your own doctors
- One of your trial team will go through this information sheet with you. Please ask us if there is anything that is not clear or if you would like more information

IMPORTANT THINGS THAT YOU NEED TO KNOW

- The REACT trial is run (sponsored) by the University of Birmingham and is funded by the National Institute of Health Research
- You have been invited to take part as you developed arthritis following Immunotherapy for cancer (Immune Checkpoint Inhibitor induced Inflammatory Arthritis; ICI-IA)
- The trial is comparing two approaches to the treatment of ICI-IA. By taking part in this trial you can help us see which approach would be better for treatment of future patients with ICI-IA
- You will be randomised onto one of two treatment 'arms' in the trial. Patients in Arm A will receive current standard of care starting with prednisolone ('steroid' tablets). Arm B will receive adalimumab without prednisolone
- Adalimumab is well-tolerated and effective in the management of other forms of inflammatory arthritis but is typically only used when other medications fail to work
- Adalimumab is a drug that blocks an inflammation causing protein known as Tumour Necrosis Factor (TNF)
- You will attend a screening visit to check if you are suitable to take part. If you are, you will be randomised into one of the two trial 'arms' and attend for the treatment visits
- Your involvement in the trial will be for approximately 48 weeks, and will require you to attend clinic 11 times. To put this in context, patients not taking part in the trial may expect to attend clinic at least 4-6 times (approximately) over a similar period for the management of their arthritis
- At selected sites, we may also ask permission to take a sample of the lining of an inflamed joint under ultrasound guidance before treatment and 12 weeks after (synovial biopsy). However, this is optional, and you can take part in the trial without having a biopsy
- You can stop taking part in this trial at any time
- In this trial we will collect information from you. This information will be handled in accordance with the UK Data Protection Legislation

CONTENTS

1. Why are we doing this trial?
2. Which treatment will I receive?
3. Why am I being asked to take part?
4. What do I need to know about the treatment
5. What will I need to do if I do take part?
6. Patient questionnaires
7. Possible symptoms & side-effects
8. Prohibited medication
9. Pregnancy and potential harm to an unborn child
10. More information about taking part
11. After the trial ends
12. Your confidentiality
13. How to contact us

HOW TO CONTACT US

If you have any questions about this trial, please talk to your local trial team:

<insert clinician> on <**insert phone number**>

1. WHY ARE WE DOING THIS TRIAL?

- We want to understand if there is a difference between treatment arms in:
  - the number of patients who are free of joint swelling after 24 weeks without having to use steroids such as prednisolone
  - how quickly patients' arthritis gets better

2. WHICH TREATMENT WILL I RECEIVE?

The treatment you receive will be chosen by a process called 'randomisation', where the treatment is randomly allocated by a computer, which is like making a choice by tossing a coin. This means you have an equal chance of being put into one of the two treatment groups. Neither you nor your trial doctor will decide which group you go into. You and your trial doctor will find out which treatment you are receiving as soon as you are randomised into a treatment 'arm' (before you start trial treatment).

The two treatments 'arms' in this trial are:

- **Arm A:** Standard of care*. This will be like the treatment you will be offered if you choose not to take part in the trial or
- **Arm B:** Adalimumab without prednisolone (steroids)

If you fail to respond to treatment in either arm, then your treatment may be changed, or additional treatments offered to you to help control your arthritis better. Any changes to your treatment will be decided by your trial doctor in conjunction with yourself and will follow local practice informed by treatment recommendations.

* initial dose of a steroid called prednisolone, 20mg/day or 40mg/day decided by the treating rheumatologist prior to randomisation

3. WHY AM I BEING ASKED TO TAKE PART?

- We are looking to recruit 70 people with ICI-IA in this trial. You have been diagnosed with ICI-IA and your doctor believes you may be suitable to take part in the trial
- Your decision to take part in this trial is entirely voluntary. If you decide not to take part, you doctor will continue to treat you with the best means available and the standard of your care will not be affected
- If you choose to take part in the trial, but later choose to withdraw, we would still like to collect information about your treatment and disease activity as this will be invaluable to our research. If you would not like this to happen, please discuss this with your doctor

- By taking part in this trial, you will help us to understand whether adalimumab (a form of anti-TNF therapy) is more effective in helping patients' arthritis improves without their having to use prednisolone (steroid tablets)
- In the long-run, we also want to also understand if early use of adalimumab may help patients to become free of arthritis and arthritis treatments. We will also collect data on the relative safety of the two treatment approaches
- By taking part you will be helping to see if Anti-TNF, in the form of adalimumab, without steroids could be used in the future as a first-line treatment for people with ICI-IA. In other words, if it should become standard of care

4. WHAT DO I NEED TO KNOW ABOUT THE TREATMENTS?

Arm A: Standard of Care

- Prednisolone is a medicine used to treat a wide range of health problems including allergies, inflammation-associated conditions and to prevent organ rejection after a transplant. It is in tablet form
- It helps by reducing swelling (inflammation) and can also calm down your immune system. This helps autoimmune conditions, like rheumatoid arthritis, where your immune system mistakenly attacks its own tissues
- Prednisolone is a 'steroid' (glucocorticoid/corticosteroid) medicine. Glucocorticoids/corticosteroids are not the same as anabolic ('body-building') steroids
- You will be allocated either 20mg/day or 40mg/day, decided by the treating rheumatologist, before randomisation
- Your dose will be reduced over the course of the trial
- Prednisolone is already used in ICI-IA patients
- If your arthritis fails to respond to prednisolone, or recurs after the dose of prednisolone is reduced, you may be offered additional medications to help control your arthritis
- One example of an additional medication is known as methotrexate. This is a once-weekly treatment given in tablet form and is a first-line treatment for other forms of inflammatory arthritis such as rheumatoid arthritis
- If these additional treatments fail to control your arthritis, you may then be offered other treatment that may include the anti-TNF drug adalimumab
- When your arthritis is fully controlled, we may consider reducing your medication to see whether your arthritis recurs
- Around two-thirds of patients have persistent arthritis and/or require ongoing treatment for arthritis in the first year after onset
- Decisions about the further management of your arthritis will be taken in conjunction with you and your Oncologist and will follow local practice informed by treatment recommendations

Arm B: Adalimumab without glucocorticoids

- Adalimumab is a drug that helps stop inflammation and is used to treat inflammatory conditions
- Adalimumab, a type of Anti-TNF, is a biological medicine. This means it is made from proteins normally produced by the body and is used to reduce swelling (inflammation) by acting on your immune system
- Adalimumab is already used in ICI-IA patients, but is typically used only when other medications have failed
- Adalimumab doses will be given by a subcutaneous injection (2 injections if your dose is increased), which is an injection into the layer of fat underneath the skin given with a short needle
- Adalimumab doses will be taken every 2 weeks and will be given in clinic to start with, then administered at home
- If the adalimumab fails to control your arthritis, you may be offered other therapies in place of adalimumab, or in addition to adalimumab
- You will receive adalimumab for the first 24 weeks. At week 24, if your arthritis has been very well controlled, we may consider stopping this. If your arthritis returns after stopping adalimumab, it may be restarted and continued through to the end of the trial at week 48

5. WHAT WILL I NEED TO DO IF I TAKE PART?

- If you decide to take part, you will be asked to sign a consent form for the REACT trial and you will be given a copy of this to keep
- You will be asked to attend additional hospital appointments in addition to your normal treatment visits. There are 13 visits over a 48 week period, although two of these may be conducted by telephone. These are detailed in the Schedule of Events on page 6
- We will check you are still happy to continue the trial at every visit

The number of visits is the same for each arm and they will consist of:

SCREENING VISIT

- You will be asked to sign a consent form for the REACT trial
- To see if you can take part, your doctor or nurse will check you are eligible by asking relevant questions and performing tests. These are detailed in the Schedule of Events on page 6. Please note that your GP will need to be informed if some results return positive
- A starting dose for prednisolone will be decided in case you are allocated standard of care treatment
- This visit can take up to 1.5 hours to complete
- This visit can take place on the same day as Week 0 (first treatment), if all needed results are available, or up to two weeks before

TREATMENT VISITS

First treatment visit - Week 0

- As part of this trial, you will attend clinic more often than your normal visits (see **Figure 1 – Visit Timeline**)
- You will be randomised to a treatment 'arm'. If you are allocated Arm B, we will show you how to administer the subcutaneous injection
- We will examine your joints to see how many are swollen and how many are tender
- You will complete the baseline Quality of Life questionnaires
- We will review your current medications and any adverse events related to your treatment
- We will take research blood samples, and routine samples if needed
- If you are at a hospital that can use ultrasound to help sample the lining of an inflamed joint (synovial biopsy), and you have agreed to this, we will take the synovial biopsy. **This is optional** (see the **page 7** for further details)
- Your first treatment will be administered
- This visit may last up to 1½ hours to complete, or up to 3 hours if it includes an optional synovial biopsy
- For details of further assessments see the Schedule of Events on page 6

Further treatment visits - Weeks 2, 4, 6, 8, 12, 16, 20, 24, 32, 40, 48

- All visits apart from Weeks 2 and 6 will be in the hospital. Weeks 2 and 6 may be conducted via telephone
- Treatment will be administered at every visit
- Your general health will be checked
- We will review your current medications and any side effects related to your treatment
- We will examine your joints to see how many are swollen and how many are tender
- At Weeks 24 and 48, a second assessor will also examine your joints. This person will be unaware of which treatment arm you have been allocated to
- We will take a single research blood sample, and routine samples if needed for your clinical care (see the Schedule of Events on **page 6** for details of the amount of blood that will be taken)
- At Week 12 we will take additional research blood samples
- At Week 12 we will take a second synovial biopsy if you have consented to this
- You will complete Quality of Life questionnaires at most visits

- Most treatment visits will last less than 45 mins. Visits at weeks 12, 24 and 48 which involve additional questionnaires or a second joint count assessor, may last longer (up to 1¼ hours). The Week 12 visit that includes an optional synovial biopsy may take up to 3 hours to complete
- For details of further assessments see the Schedule of Events on page 6

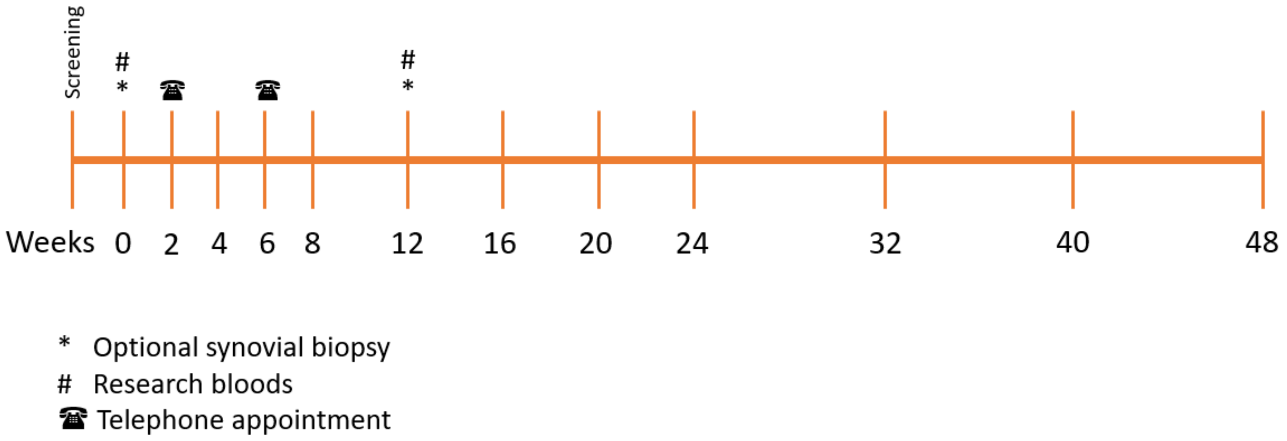

Figure 1 Visit Timeline

Schedule of Events Table

What you will be asked to do at each visit is explained below.

Visit name	Screening	Week 0	Week 2	Week 4	Week 6	Week 8	Week 12	Week 16	Week 20	Week 24	Week 32	Week 40	Week 48
Week	Week -2	Week 0	Week 2 ☎	Week 4	Week 6 ☎	Week 8	Week 12	Week 16	Week 20	Week 24	Week 32	Week 40	Week 48
Informed Consent	X												
Eligibility Assessment	X												
Randomisation	X												
Health History	X												
Physical Examination[1] (only if required for your clinical care)	X	X		X		X	X	X	X	X	X	X	X
Hepatitis and HIV test[2]	X												
Pregnancy test[3]	X		(X)	(X)	(X)	(X)	(X)	(X)	(X)	(X)	(X)	(X)	(X)
Blood tests[4] Total blood in teaspoons (approx)	4	9		1		1	8	1	1	1	1	1	1
Chest X-ray[5]	X												
Examination of joints	X	X		X		X	X	X	X	X	X	X	X
Independent examination of joints										X			X
Treatment		X	X	X	X	X	X	X	X	X	X[6]	X[5]	X[5]
Review of arthritis medications		X	X	X	X	X	X	X	X	X	X	X	X
Review of side effects related to arthritis drugs or cancer immunotherapy		X	X	X	X	X	X	X	X	X	X	X	X
Optional synovial biopsy[7]		X					X						

[1] Symptom-directed only

[2] Hepatitis and HIV at screening, if not already performed within the 6 months prior to screening.

[3] To be done at screening and the first post-treatment trial visit

[4] Additional blood tests may be requested if required as part of your clinical care

[5] Only if no chest imaging within the previous 6 months

[6] Withdrawal of treatment if in remission

[7] Week 0 biopsy can take place up to 14 days prior to the visit date. Week 0 research bloods and optional biopsy must be taken prior to trial drug administration (adalimumab or standard of care)

Health History

Your doctor will ask you questions about your previous health, wellbeing and lifestyle choices. Your doctor will ask you the details of any medications you are currently taking, and details of any previous cancer treatments. You will also be asked if your medications change during the trial.

Physical Examination

Your blood pressure will be measured at every visit. Other than your joints, we will not routinely examine other parts of your body unless this is needed for your clinical care.

Blood Tests

At the screening visit we will take blood to assess if you have been infected with certain blood-borne viruses or tuberculosis. This is done to make sure we can safely treat you. We will also measure a marker of inflammation. The volume of blood will be approximately 20 ml (4 teaspoons), but we may not need to take some of these blood tests if you have had them done in the previous 6 months. Blood samples used for research will be taken at Week 0 and Week 12. This will be approximately 45ml (9 teaspoons) at week 0 and 40ml at week 12 (8 teaspoons). At other visits we will also take 5 ml of blood (1 teaspoon) for measurement of an inflammation marker.

We may also take routine bloods at any visit if these are needed for your clinical care and have not already been done as part of your routine hospital visits or GP appointments. These will only be taken if needed. These blood tests are taken in the same way as any other normal blood test – it may cause a momentary sharp pain when the needle goes into your arm and there is a small risk of bruising.

Chest X-ray

An x-ray is a painless test which produces an image of structures in the body. A chest x-ray will be performed at the screening visit but only if you have not had a chest x-ray or CT imaging of the chest within the last 6 months.

Biopsy of joint (optional)

This joint biopsy, also called a synovial biopsy, is the removal of small pieces of tissue lining a joint under ultrasound guidance. These samples will be used in future ethically approved research and will be invaluable for helping us understand the biology of ICI-IA and therefore may help guide treatment of future patients.

Samples will be collected using a 'keyhole' procedure by a trained professional and should take no longer than 60 minutes. Ultrasound guidance makes the procedure shorter and more comfortable, using a single tiny entry point for taking samples under local anaesthetic. The ultrasound probe will remain on the skin overlying the joint to guide a needle which will be used to insert a tube no greater than 2.3 mm wide (less than 1/8th of an inch) into the outer aspect of the joint. Up to 24 very small fragments, around the size of a pinhead, will be taken. After samples have been taken, this entry point will be covered with a simple dry dressing. You will be able to walk afterwards but we recommend you are accompanied home after the procedure. If you cannot be accompanied, please discuss with your trial team before the procedure.

Examination of joints

An examination of your joints for swelling and tenderness will be carried out by a healthcare professional with training and experience in joint examination. This will assess the joints in your hands, feet, wrist, elbow, shoulder, jaw, clavicle/breastbone, hip, knee and ankle and is done on eleven visits during the trial (Screening, weeks 0, 4, 8, 12, 16, 20, 24, 32, 40, 48).

At weeks 24 and 48, a second independent assessor will also examine your joints. This person will be unaware of which treatment arm you have been allocated to.

Quality of life Patient Questionnaires

You will be asked to fill in questionnaires about your quality of life and symptoms related to your arthritis. The questionnaires are in paper form *(See Section 6)* and will be completed by you during some of your trial visits.

Symptoms & Side-Effects

At every visit your doctor or nurse will ask you if you had any symptoms of feeling unwell or possible side-effects related to your arthritis medications or to your cancer immunotherapy.

Telephone call

Week 2 and Week 6

Your trial doctor will phone you to see how you are. This allows you to speak to your doctor, but doesn't require you to travel to hospital for a visit in person. One or both of these visits could be converted to be in person if you prefer, or if you have been allocated to the adalimumab arm and require further training in self-injection.

6. PATIENT QUESTIONNAIRES: SYMPTOM AND QUALITY OF LIFE INFORMATION

Why are we assessing possible side-effects, symptoms and how you feel?

Some treatments can make people feel better, but others can cause symptoms or possible side-effects that make people feel worse. We would like you to let us know about your symptoms or possible side-effects and how you feel during the trial by completing questionnaires.

What do the questionnaires assess?

We ask that six questionnaires are completed:

A. The Patient Global Assessment is a single question which uses a visual scale to ask how your arthritis is on that particular day
B. Patient Assessment of Pain is a single question which uses a visual scale to measure average pain during the past 7 days
C. Patient Assessment of Fatigue is a single question which uses a visual scale to measure average fatigue during the past 7 days
D. The Patient Acceptable Symptom State is a single question that asks if your arthritis symptom burden is acceptable or unacceptable.
E. The Health Assessment Questionnaire – Disability Index (HAQ-DI) is a 20-item questionnaire measuring how you can function in eight different areas (dressing, arising, eating, walking, hygiene, grip and activities)
F. The EuroQol 5 Level EQ-5D Quality of Life Questionnaire (EQ-5D-5L) includes 5 tick box questions and one visual scale to assess different aspects of quality of life
G. The ICECAP-A (ICEpop CAPability measure for Adults) is a measure of well-being that asks you five questions. For each question, you need to select one out of four statements that best describes your overall quality of life at the moment.

Can someone fill in the questionnaires for me?

No, because we really want to understand your point of view we ask that you fill in the questionnaires yourself.

How often will I have to fill in questionnaires?

We ask that you fill in the questionnaires A – D (single questions) at all visits except Weeks 2 and 6. Questionnaires E – G will need to be completed at Week 0, 12, 24 and 48. This will allow us to see whether there is a difference between the treatment arms in how they make you feel.
You will be given the questionnaires to complete during your visit. They will be paper questionnaires and will take approximately 5 – 10 minutes to complete all of them.

7. POSSIBLE SYMPTOMS & SIDE-EFFECTS

- **Arm A:** Standard of care with Prednisolone

Prednisolone is an established medicine used to treat a wide range of health problems.

Undesirable effects
A wide range of side effects can be associated with prednisolone. These may include:

- Weight gain
- Indigestion
- Problems sleeping (insomnia)
- Feeling restless
- Sweating a lot
- Mild mood changes
- Increase in blood sugar
- Increase in blood pressure
- Increased risk of infection
- Easy bruising
- Thinning of bones

- **Arm B:** Adalimumab without steroids

Adalimumab is an established medicine that is usually very well-tolerated. The most commonly reported side effects are:

- Infections with symptoms of sore throat, fever, diarrhoea, coughing up green phlegm (nasopharyngitis, upper respiratory tract infection and sinusitis)

- Injection site reactions (bruising, redness, bump or itching where the needle goes into the skin. These effects could also be seen at places on your skin where you previously had an injection)
- Headache
- Musculoskeletal pain

You may be more susceptible to infections. There is a possibility of reactivation of tuberculosis (TB), although this is very rare when people are screened for previous TB infection. You should also see your doctor if you develop chickenpox, shingles or measles, or come into contact with someone who has chickenpox, shingles or measles. These illnesses can be worse if you're on adalimumab. Protein based drugs such as adalimumab may rarely cause allergic reactions. Symptoms may include difficulty in breathing, dizziness, itching, swelling of the lips, tongue or throat, coughing or rash. In general, most symptoms are manageable. Contact your healthcare team if you think this may be happening. If the reaction is very bad, then it's important you see your trial doctor or GP or go to your nearest Accident and Emergency department (A&E) as soon as possible.

The treatments in both arms work to control arthritis by suppressing inflammation. What we don't know, is whether this may also impair the immune response to your underlying cancer. There is some data to suggest that very high doses of steroid (higher than those proposed here) may be harmful, and animal model data suggesting that anti-TNF may be helpful. However, whilst a difference between treatment arms is possible, this is currently unknown. The only possible way to be more certain of the relative benefits or harms of the two treatment approaches on the underlying cancer response is to undertake a clinical trial such as this. During the course of the trial, we will collect data from your cancer doctor and from your routine cancer imaging to help us understand this further.

Side effects from tests done as part of this trial

- **Blood test:** Taking blood may cause bruising at the place where the needle goes into the skin. Fainting and, in rare cases, infection may occur
- **Synovial Biopsy (optional, and at selected sites only):** Small samples of your joint lining will be collected under ultrasound guidance. Samples will be collected using a 'keyhole' procedure and should take no longer than 60 minutes. Local anaesthetic will be used to collect this sample. The insertion of the needle and tube could cause bruising and redness. There is a small risk of infection. You will be able to walk afterwards but we recommend you are accompanied home after the procedure. The joint may be a little uncomfortable for up to two days after the procedure which can be treated with your usual painkillers. The entry point will heal leaving a tiny 2-3 mm scar (less than 1/8th of an inch) More than 5000 of these procedures have been performed worldwide and the most recent published data confirm that adverse events over and above mild discomfort occur in less than 1 in 500 procedures, with no record of infection occurring
- **Chest x-ray:** If you take part in this trial you will have a maximum of one Chest X-Ray. This scan might be extra to scans that you would have if you did not take part. This procedure uses ionising radiation to form an image of your body and/or provide treatment and/or provide your doctor with other clinical information. Ionising radiation may cause cancer many years or decades after the exposure. We are all at risk of developing cancer during our lifetime. 50% of the population is likely to develop one of the many forms of cancer at some stage during our lifetime. Taking part in this trial will add only a very small chance of this happening to you

8. PROHIBITED MEDICATION

Your trial doctor will check you are not taking any medication that is prohibited during the course of this trial. Please ask the trial doctor if you have any concerns. The prohibited medications will depend on the arm you are randomised to as follows:

- <u>**Arm A:**</u> Standard of care with Prednisolone
  - Use of protein based drugs against arthritis should be avoided in the first 12 weeks after baseline.

- <u>**Arm B:**</u> Adalimumab without steroids
  - If you are taking steroids at screening, you must not take these longer than 2 weeks prior to the baseline visit and should be discontinued at baseline on first administration of adalimumab
  - Use of protein based drugs against arthritis should not be used with adalimumab

Live vaccines should be avoided whilst you are on treatment with trial drugs or other immunosuppressive drugs.

9. PREGNANCY AND POTENTIAL HARM TO AN UNBORN CHILD

- The Summary of Product Characteristics for prednisolone advises use for prolonged periods or repeatedly in pregnancy may result in stillbirth. Cataracts have been observed in infants born to mothers treated with long term prednisolone. Prednisolone should only be prescribed when the benefits to the mother and child outweigh the risks. When treatment is essential however, patients with normal pregnancies may be treated as though they were not pregnant
- The Summary of Product Characteristics for adalimumab advises treatment should only be used in pregnancy if clearly needed. Adalimumab may cross the placenta into the serum of infants, therefore these infants may be at increased risk of infection. Administration of live vaccines (e.g., BCG vaccine) to infants exposed to adalimumab during pregnancy is not recommended for 5 months following the mother's last adalimumab injection during pregnancy
- National Institute for Health and Care Excellence (NICE) approved British Society of Rheumatology (BSR) guidance states that the treatments used within this trial (prednisolone and adalimumab) are compatible with pregnancy. Therefore, contraceptive use will not be mandated as part of this trial. However, your Oncologist may have advised you not to become pregnant if you remain on your cancer immunotherapy
- If you or your partner becomes pregnant, we will seek your/your partner's consent to collect data on the outcome of your/your partner's pregnancy from the mother's and baby's medical notes.

10. MORE INFORMATION ABOUT TAKING PART

Are there any medications I cannot take whilst I am on this trial?

This depends on which treatment arm you are randomised to.
Patients randomised to Arm A (standard of care with prednisolone), will not be allowed to take any adalimumab medication for the first 12 weeks.
Patients randomised to Arm B (adalimumab without prednisolone) will not be allowed to take any form of steroids for the arthritis from screening. If you have been taking them before screening, you may still be able to take part in the trial but will need to stop these when you start adalimumab. Steroids would be allowed if you fail to respond to adalimumab, or if needed for another indication after you have started the trial.

What will happen to my treatment when I finish the trial?

- You will return to clinical care as normal
- Your clinical team will continue to keep you informed about all treatments from clinic.
- If you have been randomised to take adalimumab and have found this helpful and wish to continue it, please discuss with your trial doctor as to whether this is possible, as local funding for this may need to be arranged.

Can I change my mind about taking part?

- Yes, you can change your mind at any point. You do not have to give a reason and your future care will not be affected.
- If you decide you do not wish to carry on with the trial, you will have the option to:
- Withdraw from the trial completely.
- Withdraw from the treatment only but carry on attending follow up visits.
- If you choose to take part in the trial, but withdraw later, we would still like to collect information about your arthritis activity and cancer response as this will be valuable to our research.
- Any information collected before you withdraw from the trial will be kept and used for final reports.
- If you would prefer that we do not collect any further information, please let your trial doctor know when you withdraw from the trial.
- Information already collected before your withdrawal from the trial will be kept and analysed.
- It is possible that your trial team may suggest you withdraw from the trial. The reason for this will be explained by your trial team and they will arrange for your normal care to continue.

What if I experience a problem whilst I am on the trial?

- If you have any concerns about any part of this trial, you should speak to your trial team who will do their best to answer your questions. Please find their contact number at the end of this form.
- If you remain unhappy and wish to make a formal complaint, you can do this through your hospital's Patient Advice and Liaison Services (PALS):
- < insert local contact details>
- In the event that something does go wrong and you are harmed during the trial there are no special compensation arrangements. If you are harmed then you may have grounds for legal action but you may have to pay your legal costs. The University of Birmingham has in place Clinical Trials indemnity coverage for this trial which provides cover to the University for harm which comes about through the University's, or its staff's, negligence in relation to the design or management of the trial and may alternatively, and at the University's discretion provide cover for non-negligent harm to participants.
- NHS Trust and Non-Trust Hospitals have duty of care to patients treated, whether or not the patient is taking part in a clinical trial and the normal NHS complaints mechanisms will still be available to you (if appropriate).
- It is possible that your trial team may suggest you withdraw from the trial. The reason for this will be explained by your trial team and they will arrange for your ongoing care.
- If you wish to complain about how your information has been used, you can contact the University's Data Protection Officer using the following email address dataprotection@contacts.bham.ac.uk

Who has reviewed this trial?

- All research in the NHS is looked at by independent group of people called a Research Ethics Committee to protect your safety, rights, wellbeing and dignity. This trial has been reviewed and given favourable opinion by the <insert Research Committee> and by the local NHS Trust Research and Development department at your hospital.
- While the trial is ongoing the results will be reviewed by an independent Data Monitoring Committee (DMC) to ensure that it is appropriate to continue with the trial.

Can my travel be reimbursed?

- When you come into hospital for your trial visits, reasonable travel expenses can be reimbursed.
- A value of up to £50 per visit can be repaid on production of receipts or other evidence of cost. Policies vary across hospitals so please speak to your research team to clarify what is needed to claim travel expenses.
- If you travel to hospital by car, travel will be reimbursed at the standard NHS patient reimbursement rate of your local NHS trust (this is typically 45 pence per mile).
- If you feel that your travel expenses are likely to be more than £50 per treatment visit, due to the distance you have to travel to the hospital, car parking charges or any other reason, then please discuss this with your trial team. Depending on individual circumstances, it may be possible to find additional ways to help with extra travel or accommodation costs.
- Food and drink may be provided during your trial visits. Please ask your trial team for details.

What will be done with the trial results?

- Results of the trial will be submitted to a medical journal, after being reviewed by other doctors. You will not be identified in any results resulting from this research.
- A lay summary, written in plain English with simple terminology will be published on the website www.isrctn.com. Your research team will be sent a plain English summary of the results which they will send to you when the trial is fully completed.

Can I still have my vaccine or booster whilst I am on the trial?

Yes, non-live vaccines or boosters are allowed. It is recommended that you are up to date on all age-appropriate vaccinations before you start the trial or as soon as possible after starting during the trial. Please discuss any planned vaccinations with your doctor.

What if new information about the effects or safety of the trial treatments becomes available when I am on the trial?

- Sometimes we get new information about the treatment being studied
- If this happens, your trial team will inform you and discuss with you whether you should continue in the trial
- If you decide not to carry on, your trial team will make arrangements for your normal care to continue
- If you decide to continue in the trial, your trial team may ask you to sign an updated consent form
- It is possible that your trial team may suggest you withdraw from the trial. The reason for this will be explained by your trial team and they will arrange for your ongoing care.

Where is the trial taking place?

This trial is currently being offered at specific hospitals in the UK.

11. YOUR CONFIDENTIALITY

All information collected about you for this trial will be subject to the General Data Protection Regulation and Data Protection Act 2018 and will be kept strictly confidential. University of Birmingham is the Sponsor for this trial and is based in the UK. The use of 'we' in the following text refers to the Sponsor i.e. 'The University of Birmingham'. We will be using information from your medical records in order to undertake this trial and will act as the data controller for this trial. This means that we are responsible for looking after your information and using it properly. The University of Birmingham and the NHS will keep identifiable information about you for at least 15 years after the trial has finished, this allows the results of the trial to be verified if needed.

All information collected by the Sponsor will be securely stored at the University of Birmingham (REACT Trial Office) on paper and electronically and will only be accessible by authorised personnel. The only people in the University of Birmingham who will have access to information that identifies you will be people who manage the trial or audit the data collection process. With your permission, your trial doctor will provide your initials and month and year of birth when they enter you into the trial and they will notify your GP that you intend to participate in the trial. They will also send a copy of your signed Informed Consent Form in the post to the REACT Trial Office.

The NHS will use your name and contact details to contact you about the research trial, and make sure that relevant information about the trial is recorded for your care, and to oversee the quality of the research.

In the REACT Trial Office you will be identified by a unique trial number. In routine communication between your hospital and the REACT Trial Office you will usually only be identified by trial number and initials. On occasion you will also be identified by your month and year of birth. Data may be provided to the REACT Trial Office on paper or electronically.

By taking part in the trial you will be agreeing to allow research staff at your hospital, and staff from the REACT Trial Office, to look at the trial records, including your medical records. It may be necessary to allow authorised personnel from The University of Birmingham, government regulatory agencies (e.g. Medicines and Healthcare products Regulatory Agency [MHRA]), the Sponsor and/or NHS bodies to have access to information about you. This is to ensure that the trial is being conducted to the highest possible standards.

In addition, if you have provided blood and biopsy research samples for the trial, your trial number will be passed on to personnel at external (to your local hospital) research laboratories to help them identify the blood and biopsy samples. With your consent, these samples will be stored for future ethically approved research to help us understand ICI-IA and its response to treatment.

All individuals who have access to your information have a duty of confidentiality to you. Under no circumstances will you be identified in any way in any report, presentation or publication arising from this trial.

If you choose to withdraw from the trial treatment, we would still like to collect relevant information about your health, as this will be invaluable to our research. If you have any objection to this, please let your trial doctor know.

You can withdraw your consent to our processing of your data at any time. Your rights to access change or move your information are limited, as we need to manage your information in specific ways in order for the research to be reliable and accurate. If you withdraw from the trial, we will keep the information about you that we have already obtained, including any samples that have already been taken up until the date you withdraw. To safeguard your rights, we will use the minimum personally-identifiable information possible. Under the provisions of the General Data Protection Regulation and Data Protection Act 2018 you have the right to know what information the REACT Trial Office have recorded about you. If you wish to view this information or find more about how we use this information, please contact Legal Services at the address below. Please note that a small fee may be payable to retrieve this information.

Legal Services
University of Birmingham
Edgbaston
Birmingham, B15 2TT

You can find out more about how we use your information in our Privacy Policy on our website www.birmingham.ac.uk/crctu.

12. HOW TO CONTACT US

If you have any questions or concerns about your arthritis or this clinical trial, please discuss them with your doctor. You can get in touch with the doctors and nurses at any time to discuss any doubts or worries you may have about the trial, and we will give you a card with contact details. Contact details are also shown below:

**Trial Doctor:**	<insert name>	**Telephone:**	<insert number>
**Research Nurse(s):**	<insert name>	**Telephone:**	<insert number>
**Hospital Details:**	<insert name and address of hospital>		

Thank you for taking the time to read this information

REACT

Appendix 4. Exemplar informed consent form

<To be printed on local hospital headed paper>

REACT

Informed Consent Form

Treating joints affected by Arthritis following Immunotherapy

Site: .... **Patient Trial Number:** [ ][ ]

Principal Investigator: .... **Trial Reference Number:** IRAS 1009034

Please initial each box

1. I confirm that I have read and understand the Patient Information Sheet (version .... dated..........................) for the above trial. I have had the opportunity to consider the information, ask questions and have had these answered satisfactorily. [ ]

2. I understand that my participation is voluntary and that I am free to withdraw at any time without giving any reason, without my medical care or legal rights being affected. [ ]

3. I give permission for my personal details, including name and month and year of birth, as well as a copy of this consent form to be given to the Trials Office once I am randomly allocated to a treatment arm on the trial. [ ]

4. I understand that relevant sections of my medical notes and data collected during the trial may be looked at by individuals from the Trials Office, regulatory authorities, Sponsors and/or NHS bodies, where it is relevant to my taking part in this research. I give permission for these individuals to have access to my records. [ ]

5. I understand that anonymised data from the trial may be provided to other 3rd parties (e.g. academic institutions or other pharmaceutical companies) for research, safety monitoring or licensing purposes. I understand that this data may be transmitted to countries (including the United States of America) which may have a different level of data protection to that in the UK. I understand this information will be held in a confidential manner. [ ]

6. I agree to the collection of research blood samples for future ethically approved research into arthritis and its treatment. I understand that this might involve sending the samples to laboratories within the United Kingdom and also outside the European Economic area, (including the United States of America), to countries which may have a different level of data protection to that in the UK to be stored and analysed. The information accompanying these samples will be personally unidentifiable to me (meaning it will not contain patient specific identifiable information such as name and hospital number) and my identity will remain anonymous. In addition, I understand that I will not be personally informed of the results of these specialised / non-NHS standard tests. I understand this information will be held in a confidential manner. [ ]

7. I agree to my GP being informed of my participation in this trial. ☐

8. I give consent that my GP and specialist doctors involved in my healthcare may be contacted and access given to my medical notes held by my GP. ☐

9. I give consent for my GP to provide information from my medical notes to my trial team, if requested. ☐

10. I understand that the Trials Office, may access information held by Cancer Registries and/or the NHS Digital to keep in touch with me and to follow up on my health status. ☐

11. I understand that if I withdraw from the trial, any samples and data that have been collected up to the date of my withdrawal will be analysed and used as part of the research. ☐

12. I agree to take part in the REACT trial. ☐

The following are optional and will not affect entry into the trial, please initial in each box:

	**No**	**Yes**
OPTIONAL – I agree to the collection, storage and DNA analysis of an additional blood sample for use in research associated with this trial. And I also consent for any remaining samples being used for future research purposes.	☐	☐
OPTIONAL – I agree to the collection of synovial tissue, and consent to these samples being used in future ethically approved research into arthritis and its treatment. I understand that this may involve sending the samples to laboratories in the United Kingdom and also outside the European Economic area to countries (including the United States of America) which may have a different level of data protection to that in the UK to be stored and analysed. The information accompanying these samples will be personally unidentifiable to me (meaning it will not contain patient specific identifiable information such as name and hospital number) and my identity will remain anonymous. In addition, I understand that I will not be personally informed of the results of these specialised / non-NHS standard tests. I understand this information will be held in a confidential manner.	☐	☐

______________________________	____________________	__________________________
**Name of patient**	**Date**	**Signature**

______________________________	____________________	__________________________
**Name of person taking consent**	**Date**	**Signature**

You must have signed the Site Signature & Delegation Log

Appendix 5. Guidance on management of inadequate response

Arm A – Standard of care

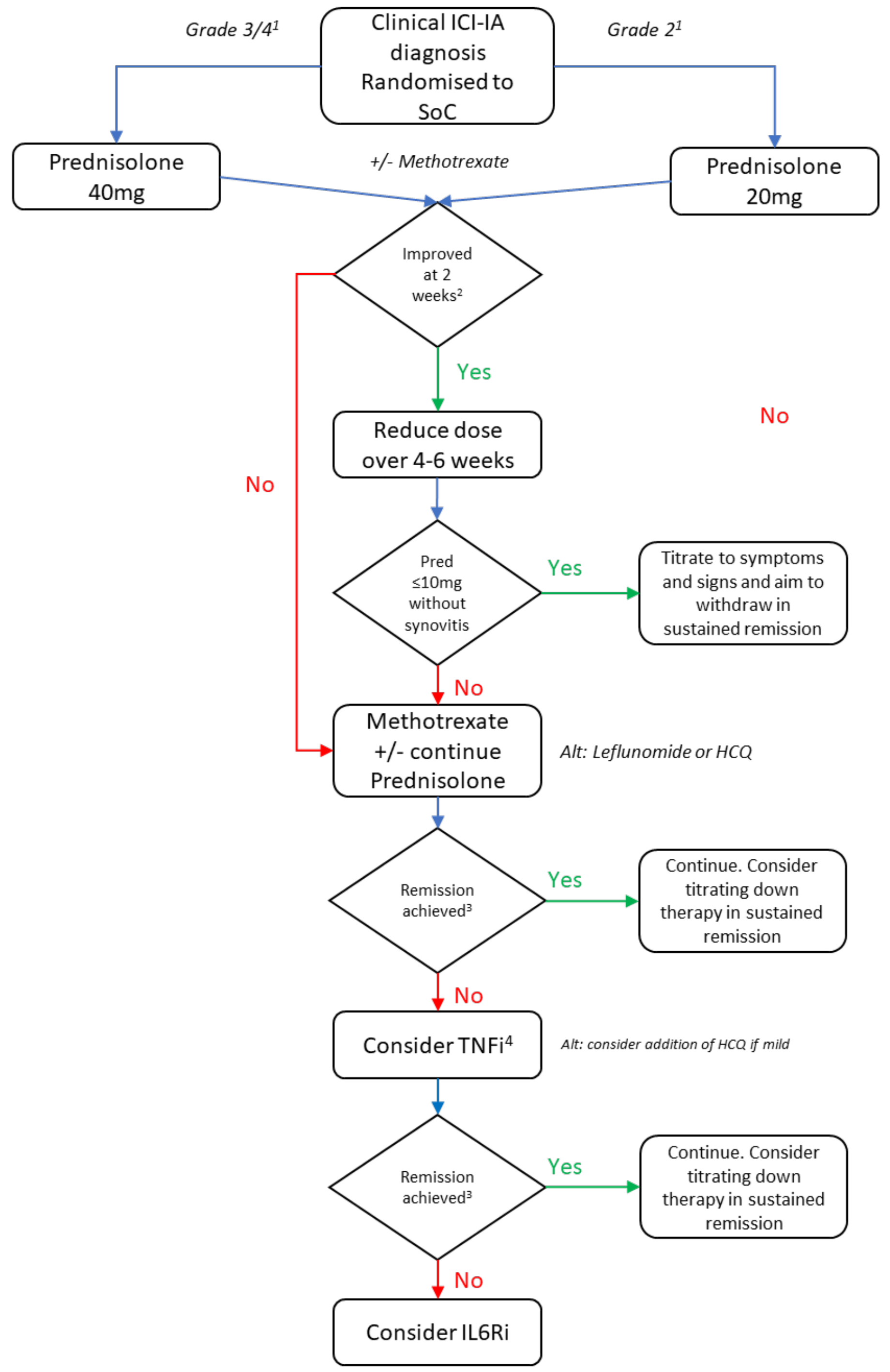

Notes:
[1]Grade 2: Moderate pain associated with signs of inflammation, erythema, or joint swelling, limiting instrumental ADL; Grade 3/4: Severe pain associated with signs of inflammation, erythema, or joint swelling; irreversible joint damage; disabling; limiting self-care ADL
[2]Subjective improvement
[3]Typically evaluated after 3 months of DMARD
[4]Local funding. Avoid biologic in first 3 months.

ADL, Activities of Daily Living. HCQ, hydroxychloroquine. DMARD, disease-modifying anti-rheumatic drug; IL6Ri, IL6 receptor inhibitor (anti-IL6R) e.g. tocilizumab or sarilumab. SoC, Standard of Care. TNFi, TNF inhibitor (anti-TNF) i.e. adalimumab.

Arm B – Adalimumab without glucocorticoids

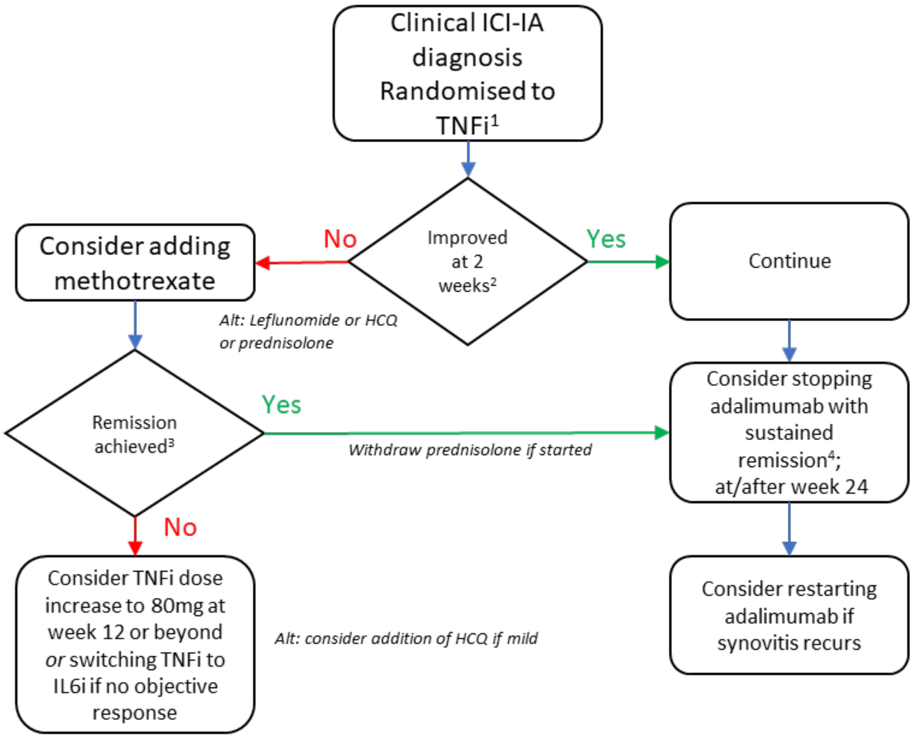

Notes:
[1]Stop steroids when starting adalimumab, if receiving these prior to starting adalimumab
[2]Subjective improvement
[3]Typically evaluated after 3 months of DMARD. With disease control, therapy will be titrated down to the minimum amount required to control synovitis if drug cessation not possible.
[4]Absence of synovitis on clinical examination on at least 2 consecutive occasions 4 weeks apart

HCQ, hydroxychloroquine. DMARD, disease-modifying anti-rheumatic drug. IL6Ri, IL6 receptor inhibitor (anti-IL6R) e.g. tocilizumab or sarilumab. TNFi, TNF inhibitor (anti-TNF) i.e. adalimumab.

The algorithms above provide recommendations for the management of inadequate response in the standard of care prednisolone (Arm A) or adalimumab without glucocorticoids (Arm B), with a treatment target of remission. Given the complex nature of immune checkpoint inhibitor-induced inflammatory arthritis (ICI-IA) care, actual management is at the discretion of the investigator in conjunction with the treating oncologist and patient, with the exception that patients randomised to receive standard of care (Arm A) should start prednisolone at 20mg or 40mg as determined prior to randomisation, and patients randomised to receive adalimumab without glucocorticoids (Arm B) should start adalimumab without glucocorticoids, and if taking glucocorticoids at screening, these should be discontinued on starting adalimumab.

Appendix 6. Secondary and exploratory outcome measures

Secondary outcome measures

1. Time to remission defined as time from randomisation to first absence of synovitis on clinical examination at Weeks 0, 4, 8, 12, 16, 20, 24, 32, 40, 48. This outcome will be calculated using Kaplan-Meier methods and differences between arms will be compared using Cox models adjusted for stratification factors. Participants who do not experience an event will be censored at their date last seen and known to be event free. Kaplan-Meier plots will be produced with 95% CIs, with median survival time and 6 month and 1 year survival rates reported, each with 95% CIs.
2. Arthritis remission measured as absence of synovitis at 24 and 48 weeks. This outcome will be summarised as the number of participants in each category, and the percentage this represents of the total evaluable population.
3. Drug-free arthritis remission measured as absence of synovitis with no immune checkpoint inhibitor-induced inflammatory arthritis (ICI-IA) treatment in the previous 4 weeks at 48 weeks. This outcome will be summarised as the number of participants in each category, and the percentage this represents of the total evaluable population.
4. 66/68 swollen and tender joint counts, defined as the number of joints that are swollen and the number of joints that are tender at Screening, Weeks 0, 4, 8, 12, 16, 20, 24, 32, 40, 48. Swollen and tender joint counts will be summarised separately. This outcome will be summarised using repeated measures plots and either means and standard deviations, or medians and interquartile ranges (dependent on observed distribution), by assessment timepoint.
5. Patient arthritis disease activity global visual analogue scale (VAS) (0-100) at Weeks 0, 4, 8, 12, 16, 20, 24, 32, 40, 48. The participant VAS is a 10cm line in which participants mark to rate their perceived health in the past week, with the left-most side being best and the right-most side being worst. The mark is measured in millimetres to provide a value and will be analysed as a continuous variable. This outcome will be summarised using repeated measures plots and either means and standard deviations, or medians and interquartile ranges (dependent on observed distribution), by assessment timepoint.
6. Physician arthritis disease activity global VAS (0-100) at Weeks 0, 4, 8, 12, 16, 20, 24, 32, 40, 48. The physician VAS is a 10cm line in which physicians mark to rate the participants health in the past week, with the left-most side being best and the right-most side being worst. The mark is measured in millimetres and will be analysed as a continuous variable. This outcome will be summarised using repeated measures plots and either means and standard deviations, or medians and interquartile ranges (dependent on observed distribution), by assessment timepoint.
7. Fatigue VAS (0-100) at Weeks 0, 4, 8, 12, 16, 20, 24, 32, 40, 48. Participants are asked to rate their average fatigue in the past week on a 100mm VAS, where a larger value indicates worse average fatigue. The fatigue VAS will be analysed as a continuous variable. This outcome will be summarised using repeated measures plots and either means and standard deviations, or medians and interquartile ranges (dependent on observed distribution), by assessment timepoint.
8. Pain VAS (0-100) at Weeks 0, 4, 8, 12, 16, 20, 24, 32, 40, 48. Participants are asked to rate their average pain in the past week on a 100mm VAS, where a larger value indicates worse average fatigue. The fatigue VAS will be analysed as a continuous variable. This

outcome will be summarised using repeated measures plots and either means and standard deviations, or medians and interquartile ranges (dependent on observed distribution), by assessment timepoint.

9. Cumulative exposure to glucocorticoids over 24 and 48 weeks, where a participants' cumulative dose of glucocorticoids will be calculated from their treatment start to 24 weeks, and from treatment start to 48 weeks. This outcome will be summarised as either mean and standard deviation, or median and inter-quartile range (dependent on observed distribution) by treatment arm.
10. Cumulative exposure to anti-TNF (or other biological DMARD or targeted synthetic DMARD) over 24 and 48 weeks, where a participants' cumulative exposure to anti-TNF or targeted synthetic DMARD (including JAK inhibitors) treatment will be calculated from their treatment start to 24 weeks, and from treatment start to 48 weeks. This outcome will be summarised as either mean and standard deviation, or median and inter-quartile range (dependent on observed distribution) by treatment arm.
11. Cumulative exposure to other conventional synthetic DMARD over 24 and 48 weeks, where a participants' cumulative exposure to synthetic DMARD not included above will be calculated from their treatment start to 24 weeks, and from treatment start to 48 weeks. This outcome will be summarised as either mean and standard deviation, or median and inter-quartile range (dependent on observed distribution) by treatment arm.
12. Cumulative exposure to ICI over 24 and 48 weeks, where a participants' cumulative exposure to any ICI will be calculated from their treatment start to 24 weeks, and from treatment start to 48 weeks. This outcome will be summarised as either mean and standard deviation, or median and inter-quartile range (dependent on observed distribution) by treatment arm.
13. Adverse events related to arthritis interventions using CTCAE version 5.0 [1] at Weeks 0, 4, 8, 12, 16, 20, 24, 32, 40, 48. This outcome will be summarised as the number of participants in each category, and the percentage this represents of the total evaluable population.
14. Serious adverse events using CTCAE version 5.0 [1] at Weeks 0, 4, 8, 12, 16, 20, 24, 32, 40, 48. This outcome will be summarised as the number of participants in each category, and the percentage this represents of the total evaluable population.
15. Other immune-related adverse events (number of organs and toxicity level) using CTCAE version 5.0 [1] at Weeks 0, 4, 8, 12, 16, 20, 24, 32, 40, 48. This outcome will be summarised as the number of participants in each category, and the percentage this represents of the total evaluable population.
16. Functional status as assessed by the Health Assessment Questionnaire Disability Index (HAQ-DI) [2] at Weeks 0, 12, 24, 48 and reported on a continuous scale from 0 (indicating best functional status), to 3 (indicating worst functional status). This outcome will be summarised using repeated measures plots and either means and standard deviations, or medians and interquartile ranges (dependent on observed distribution), by assessment timepoint
17. Health-related quality of life as assessed by EQ-5D-5L questionnaire [3] at Weeks 0, 12, 24, 48 and reported as a code containing the reported function level for each of the five health dimensions ranging from 11111 to 55555 corresponding to best health and worst health, respectively. This outcome will be summarised using repeated measures plots and either means and standard deviations, or medians and interquartile ranges (dependent on observed distribution), by assessment timepoint.

18. Well-being as assessed by ICECAP-A questionnaire [4] at Weeks 0, 12, 24, 48 where participant responses for each section are assigned UK tariff values, where a higher value indicates better functionality. The sum of the values for each section are used as a measure of the participants' overall wellbeing. This outcome will be summarised using repeated measures plots and either means and standard deviations, or medians and interquartile ranges (dependent on observed distribution), by assessment timepoint.
19. Investigator-assessed cancer status as complete response, partial response, stable disease, or disease progression at Weeks 0, 4, 8, 12, 16, 20, 24, 32, 40, 48. The assessment will be comprised of one or more the following: recent imaging, recent oncological correspondence, blood test results, and clinical evaluation of the patient. . This outcome will be summarised as the number of participants in each category, and the percentage this represents of the total evaluable population.
20. Cancer response at weeks 24 and 48 as defined by Response Evaluation Criteria in Solid Tumors (RECIST) version 1.1 [5]. This outcome will be summarised as the number of participants in each category, and the percentage this represents of the total evaluable population.
21. Overall survival defined as the length time from participant randomisation to death, collected at Weeks 0, 4, 8, 12, 16, 20, 24, 32, 40, 48. This outcome will be calculated using Kaplan-Meier methods and differences between arms will be compared using Cox models adjusted for stratification factors. Participants who do not experience an event will be censored at their date last seen and known to be event free. Kaplan-Meier plots will be produced with 95% CIs, with median survival time and 6 month and 1 year survival rates reported, each with 95% CIs.
22. Progression-free survival of cancer defined as the length of time from participant randomisation to progression (defined as first occurrence of disease progression via cancer response using RECIST version 1.1 or investigator-assessed cancer status), or death at Weeks 0, 4, 8, 12, 16, 20, 24, 32, 40, 48. This outcome will be calculated using Kaplan-Meier methods and differences between arms will be compared using Cox models adjusted for stratification factors. Participants who do not experience an event will be censored at their date last seen and known to be event free. Kaplan-Meier plots will be produced with 95% CIs, with median survival time and 6 month and 1 year survival rates reported, each with 95% CIs.

Exploratory outcome measures

1. American College of Rheumatology (ACR)/European Alliance of Associations for Rheumatology (EULAR) remission criteria defined as composite outcome incorporating the swollen and tender joint counts, c-reactive protein concentrations (mg/L) and participant global assessment, and measured at Weeks 0, 2, 8, 12, 16, 20, 24, 32, 40, and 48. [6] This outcome will be assessed twice, once with 66/68 joint counts, and then again for 28 joint counts to determine if there are differences when using simpler joint counts. The number proportion of participants who meet the EULAR remission criteria will be calculated for each arm and assessment timepoint.
2. Disease activity score-28 (DAS28) defined as a composite score incorporating the 28 swollen and 28 tender joint counts (SJC28, TJC28) c-reactive protein concentration (CRP; mg/L), and participant's global assessment on a VAS from 0 to 100 (mm), and measured at Weeks 0, 2, 8, 12, 16, 20, 24, 32, 40, and 48. [7] This outcome will be summarised as either mean and standard deviation, or median and inter-quartile range (dependent on

observed distribution) by treatment arm. The number proportion of participants who meet the different DAS28 disease activity categories will be calculated for each arm and assessment timepoint.

3. Clinical Disease Activity Index (CDAI) defined as a composite of the participant global assessment and physician global assessment on a 0-10 scale (cm) and SJC28 and TJC28, and measured at Weeks 0, 2, 8, 12, 16, 20, 24, 32, 40, and 48 [8]. This outcome will be summarised as either mean and standard deviation, or median and inter-quartile range (dependent on observed distribution) by treatment arm and assessment timepoint.
4. Disease Activity in PsA (DAPSA) defined as a composite of the swollen joint and tender joint counts, pain VAS, participant global assessment and CRP (mg/dL), and measured at Weeks 0, 2, 8, 12, 16, 20, 24, 32, 40, and 48. [9] This outcome will be summarised as either mean and standard deviation, or median and inter-quartile range (dependent on observed distribution) by treatment arm and assessment timepoint.
5. Patient-centred ranked composite outcome will include oncological, rheumatological, and quality of life outcomes in a single scale. The content and development of which will be described fully in a separate publication, however it will use data collected at screening, Weeks 0, 2, 4, 6, 8, 12, 16, 20, 24, 32, 40, and 48. This outcome will be summarised using repeated measures plots and either means and standard deviations, or medians and interquartile ranges (dependent on observed distribution), by assessment timepoint.
6. Composite immune-related adverse event score comprising number of organs and grade of toxicity over time, comprising number of organs affected, number of IrAEs with a CTCAE score ≥3, and a cumulative CTCAE score will be calculated for each participant. These composite scores will be measured using CTCAE version 5.0 [1] at Weeks 0, 4, 8, 12, 16, 20, 24, 32, 40, 48, as well as a cumulative score for each participant at 24 and 48 weeks. This outcome will be summarised as either mean and standard deviation, or median and inter-quartile range (dependent on observed distribution) by treatment arm and assessment timepoint.
7. If any pregnancies occur during the trial, data on pregnancy outcomes will be collected at screening, Weeks 0, 2, 4, 6, 8, 12, 16, 20, 24, 32, 40, and 48 and reported by treatment arms using descriptive analysis.

References

1. National Cancer Institute (NCI) Common Terminology Criteria for Adverse Events (CTCAE), version 5.0. 2017. Available at: https://dctd.cancer.gov/research/ctep-trials/for-sites/adverse-events. Accessed 12-Aug-2025.
2. Seror R, Tubach F, Baron G, et al. Measure of function in rheumatoid arthritis: individualised or classical scales? *Ann Rheum Dis*. 2010;69(1):97-101. DOI: 10.1136/ard.2008.102137
3. Buchholz I, Janssen MF, Kohlmann T, et al. A Systematic Review of Studies Comparing the Measurement Properties of the Three-Level and Five-Level Versions of the EQ-5D. *PharmacoEconomics*. 2018;36(6):645-661. DOI: 10.1007/s40273-018-0642-5
4. Al-Janabi H, N Flynn T, Coast J. Development of a self-report measure of capability wellbeing for adults: the ICECAP-A. *Quality of Life Research*. 2012;21(1):167-176. DOI: 10.1007/s11136-011-9927-2
5. Eisenhauer EA, Therasse P, Bogaerts J, et al. New response evaluation criteria in solid tumours: Revised RECIST guideline (version 1.1). *European Journal of Cancer*. 2009;45(2):228-247. DOI: https://doi.org/10.1016/j.ejca.2008.10.026

6. Studenic P, Aletaha D, de Wit M, et al. American College of Rheumatology/EULAR Remission Criteria for Rheumatoid Arthritis: 2022 Revision. *Arthritis Rheumatol*. 2023;75(1):15-22. DOI: 10.1002/art.42347
7. Prevoo MLL, Van'T Hof MA, Kuper HH, et al. Modified disease activity scores that include twenty-eight-joint counts development and validation in a prospective longitudinal study of patients with rheumatoid arthritis. *Arthritis & Rheumatism*. 1995;38(1):44-48. DOI: https://doi.org/10.1002/art.1780380107
8. Aletaha D, Nell VPK, Stamm T, et al. Acute phase reactants add little to composite disease activity indices for rheumatoid arthritis: validation of a clinical activity score. *Arthritis Research & Therapy*. 2005;7(4):R796. DOI: 10.1186/ar1740
9. Schoels MM, Aletaha D, Alasti F, et al. Disease activity in psoriatic arthritis (PsA): defining remission and treatment success using the DAPSA score. *Ann Rheum Dis*. 2016;75(5):811-818. DOI: 10.1136/annrheumdis-2015-207507

Appendix 7. Analyses of secondary and exploratory measures

Secondary outcome measures

Time to event outcomes, including time to remission; overall survival (defined as the time from registration to death); progression-free survival (defined as the time from registration to first clinical progression, defined as cancer status progression, or patient death) will be calculated using Kaplan-Meier methods and differences between arms will be compared using Cox models adjusted for stratification factors. Participants who do not experience an event will be censored at their date last seen. Kaplan-Meier plots will be produced with 95% confidence intervals (CIs), with median survival time and 6 month and 1 year survival rates reported, each with 95% CIs. A log-rank test will be performed to test for statistical significance.

Binary outcomes (including arthritis remission at 24 and 48 weeks; drug-free arthritis remission at 48 weeks) will be compared between arms using Pearson's chi-squared test or Fisher's exact test, if data is normally or non-normally distributed, respectively.

Continuous, or pseudo-continuous outcomes (including cumulative exposure to glucocorticoids over 24 and 48 weeks; cumulative exposure to anti-tumour necrosis factor (TNF) or other biological disease-modifying anti-rheumatic drug (DMARD) or targeted-synthetic DMARD over 24 and 48 weeks; cumulative exposure to other conventional synthetic DMARD over 24 and 48 weeks; Cumulative exposure to any immune checkpoint inhibitor (ICI) over 24 and 48 weeks;) will be analysed by comparing mean/median values between each arm at the relevant time points. Statistical significance of a difference between treatment arms will be assessed using a t-test if data are normally distributed, and a Mann-Whittney U test if data are non-normally distributed. Continuous, or pseudo-continuous outcomes where more the outcome is collected at multiple timepoints (including: 66/68 swollen and tender joints count; fatigue visual analogue scale (VAS); patient arthritis disease activity global VAS; physician arthritis disease activity global VAS; pain VAS; HAQ-DI functional status scores) will be assessed for differences between arms using nested random effects models using linear patient trajectories through time (alternative analytical functions of time e.g., $Time^2$, or $\sqrt{Time}$ may be considered as necessary). These models will adjust for stratification factors and transformations of the dependant variable will be considered as necessitated by the observed distribution of such data.

Ordinal outcomes (including: investigator assessed cancer status; cancer status as defined by Response Evaluation Criteria in Solid Tumors (RECIST) version 1.1 [1]) will be compared between treatment arms using ordered logistic regression models, adjusted for stratification factors. A likelihood ratio test (LRT) will be used to assess the statistical significance of any difference present between the models.

Adverse events related to the arthritis interventions and serious adverse events will be analysed by comparing the number and proportion of participants who experience these outcomes by treatment arm.

Other immune-related adverse events (number of organs and toxicity level) – the number of organs and number and proportion of participants affected will be compared between arms, as will toxicity level with median cumulative and median highest toxicity level experienced compared by arm, with a t-test or Mann-Whittney U test performed to assess differences between arms, dependent on the normality of data distribution.

Health-related quality of life as assessed by ED-5D-5L will be analysed by converting health codes for each participants to a corresponding index value using weights pre-defined for participants in England. The resulting index will range from -0.59 to 1, and values will be analysed as detailed above for continuous outcomes with multiple timepoints of data collection.

Well-being as assessed by ICECAP-A will be analysed using the sum of scoring tariffs relating the answers patients give. Values will be analysed as detailed above for continuous outcomes with multiple timepoints of data collection.

Exploratory outcome measures

Data pertaining to exploratory outcome measures will be transformed as per user manuals and standard practice to derive clinically meaningful indices where relevant. These will be summarised descriptively and graphically using methods appropriate to the data type. For values with multiple domains, each domain will be tabulated with proportions for each activity level within the domains. Data regarding the pregnancy outcome will include the number of confirmed pregnancies, the duration from treatment start and most recent treatment to pregnancy confirmation and outcome, and the outcome of the pregnancy, with data separated by treatment arm. Analyses of exploratory outcomes will be with the intention of generating hypotheses for future clinical trials with significance testing performed as necessary and appropriate.

Given the patient population, it is critical to ensure that improvements in arthritis outcomes do not come at the detriment of oncology outcomes. Considering this, one of our exploratory outcomes is a patient-centred ranked composite outcome where we can classify each participants' overall outcome accounting for cancer and arthritis outcomes.

The patient-centred ranked composite outcome analysis will classify each participant's overall outcome, based on at least mortality, oncology status, arthritis status, and quality of life, into an ordinal scale. The ranking of outcome categories will be determined by consensus among the investigators and a group of patients during the conduct of the trial and will be reviewed (and potentially modified) by a sample of clinicians and patients. A full description of the outcome will be included in the statistical analysis plan (SAP) available from the REACT Trial Office (REACT@trials.bham.ac.uk). The ordinal overall outcome measure will be used to compare the randomised groups, using pseudo-continuous or ordinal regression models (depending on the classification). The main advantages of this approach are that it is more relevant to patients because it considers participants' overall outcome, incorporates multiple assessments, and it enables all participants' outcomes to contribute to the analysis. This differs from conventional analysis, which generally assesses risk: benefit on each domain independently with no way of handling conflicting conclusions from different outcomes.

References

1. Eisenhauer EA, Therasse P, Bogaerts J, et al. New response evaluation criteria in solid tumours: Revised RECIST guideline (version 1.1). *European Journal of Cancer*. 2009;45(2):228-247. DOI: https://doi.org/10.1016/j.ejca.2008.10.026

Appendix 8. Adverse event definitions

Adverse event (AE)

Any untoward medical occurrence in a clinical trial participant administered a medicinal product and which does not necessarily have a causal relationship with this treatment.

Comment:

An AE can therefore be any unfavourable and unintended sign (including abnormal laboratory findings), symptom or disease temporally associated with the use of an investigational medicinal product, whether or not related to the investigational medicinal product.

Adverse reaction (AR)

All untoward and unintended responses to an IMP related to any dose administered.

Comment:

An AE judged by either the reporting Investigator or Sponsor as having causal relationship to the IMP qualifies as an AR. The expression reasonable causal relationship means to convey in general that there is evidence or argument to suggest a causal relationship.

Serious adverse event (SAE)

Any untoward medical occurrence or effect that at any dose:

- Results in death
- Is life threatening*
- Requires hospitalisation** or prolongation of existing inpatient hospitalisation
- Results in persistent or significant disability or incapacity
- Is a congenital anomaly/birth defect
- Or is otherwise considered medically significant by the Investigator***

Comments:

The term severe is often used to describe the intensity (severity) of a specific event. This is not the same as serious, which is based on participants/event outcome or action criteria.

* Life threatening in the definition of a Serious Adverse Event refers to an event in which the participant was at risk of death at the time of the event; it does not refer to an event that hypothetically might have caused death if it were more severe.

**Hospitalisation is defined as an unplanned, formal inpatient admission, even if the hospitalisation is a precautionary measure for continued observation. Thus, hospitalisation for protocol treatment (e.g., line insertion), elective procedures (unless brought forward because of worsening symptoms) or for social reasons (e.g., respite care) are not regarded as an SAE.

*** Medical judgment should be exercised in deciding whether an Adverse Event is serious in other situations. Important Adverse Events that are not immediately life threatening or do not result in death or hospitalisation but may jeopardise the participant or may require intervention to prevent one of the other outcomes listed in the definition above, should be considered serious.

Serious adverse reaction (SAR)

An Adverse Reaction which also meets the definition of a Serious Adverse Event.

Suspected unexpected serious adverse reaction (SUSAR)

A Serious Adverse Reaction that is unexpected i.e., the nature, or severity of the event is not consistent with the applicable product information.

A Suspected Unexpected Serious Adverse Reaction should meet the definition of an Adverse Reaction, Unexpected Adverse Reaction and Serious Adverse Reaction.

Unexpected adverse reaction (UAR)

An Adverse Reaction, the nature or severity of which is not consistent with the Reference Safety Information.

When the outcome of an Adverse Reaction is not consistent with the Reference Safety Information, the Adverse Reaction should be considered unexpected.
