# Supplementary material for: Pragmatic, open-label, multicentre, randomised controlled trial to guide initial therapy for immune checkpoint inhibitor-induced inflammatory arthritis comparing standard of care (prednisolone) to adalimumab without glucocorticoids: REACT trial protocol
Source: BMJ Open. 2026 Mar 3;16(3):e116847. doi: 10.1136/bmjopen-2026-116847 (PMC12958871; doi:10.1136/bmjopen-2026-116847)
Supplement: Supplementary file 2 [file bmjopen-2026-116847.reviewer_comments.pdf]

PEER REVIEW HISTORY

BMJ Open publishes all reviews undertaken for accepted manuscripts. Reviewers are asked to complete a checklist review form and are provided with free text boxes to elaborate on their assessment. These free text comments are reproduced below.

ARTICLE DETAILS

Title (Provisional)

REACT trial protocol: A pragmatic, open-label, multicentre, randomised controlled trial to guide initial therapy for immune checkpoint inhibitor-induced inflammatory arthritis comparing standard of care (prednisolone) to adalimumab without glucocorticoids

Authors

Fisher, Benjamin; Rowe, Anna; Wilkhu, Manpreet; Williams, Emily; Turner, Elliot; Allard, Andrew; Blake, Tim; Bombardieri, Michele; Cope, Andrew; Dubey, Shirish; Mankia, Kulveer Singh; Malley, Tamir; Moore, Owen; Payne, Miranda; Plummer, Ruth; Tilby, Michael; Tillett, Tania; Wong, Ernest; Wu, Yin; Filer, Andrew; Pratt, Arthur; Pallan, Lalit; Homer, Victoria

---

This article was not externally reviewed at BMJ Open. Protocols that have been independently assessed prior to submission to BMJ Open are usually fast-tracked to publication on the grounds that further substantial changes will not be possible. This independent assessment will usually be external, independent review for both a substantial grant award from a non-commercial or government funder and ethics approval.
